# Phenotypic profiling of solute carriers characterizes serine transport in cancer

**DOI:** 10.1038/s42255-023-00936-2

**Published:** 2023-12-08

**Authors:** Vasileios Papalazarou, Alice C. Newman, Alejandro Huerta-Uribe, Nathalie M. Legrave, Mattia Falcone, Tong Zhang, Lynn McGarry, Dimitris Athineos, Emma Shanks, Karen Blyth, Karen H. Vousden, Oliver D. K. Maddocks

**Affiliations:** 1 School of Cancer Sciences, Wolfson Wohl Cancer Research Centre, University of Glasgow, Glasgow, UK; 2 Francis Crick Institute, London, UK; 3 Cancer Research UK Beatson Institute, Glasgow, UK; 4 Present Address: Metabolomics Platform, Luxembourg Institute of Health, Department of Cancer Research, Strassen, Luxembourg; 5 Present Address: Division of Oncogenomics, The Netherlands Cancer Institute, Amsterdam, The Netherlands; 6 Present Address: Novartis Institutes for Biomedical Research, Shanghai, China

**Keywords:** Cancer metabolism, Metabolomics, Metabolism

## Abstract

Serine is a vital amino acid in tumorigenesis. While cells can perform de novo serine synthesis, most transformed cells rely on serine uptake to meet their increased biosynthetic requirements. Solute carriers (SLCs), a family of transmembrane nutrient transport proteins, are the gatekeepers of amino acid acquisition and exchange in mammalian cells and are emerging as anticancer therapeutic targets; however, the SLCs that mediate serine transport in cancer cells remain unknown. Here we perform an arrayed RNAi screen of SLC-encoding genes while monitoring amino acid consumption and cell proliferation in colorectal cancer cells using metabolomics and high-throughput imaging. We identify SLC6A14 and SLC25A15 as major cytoplasmic and mitochondrial serine transporters, respectively. We also observe that SLC12A4 facilitates serine uptake. Dual targeting of SLC6A14 and either SLC25A15 or SLC12A4 diminishes serine uptake and growth of colorectal cancer cells in vitro and in vivo, particularly in cells with compromised de novo serine biosynthesis. Our results provide insight into the mechanisms that contribute to serine uptake and intracellular handling.

## Abstract (web summary)

In this study, Papalazarou et al. screen the solute carrier family and identify candidates involved of serine transport in colorectal cancer cells. They further characterize cytosolic SLC6A14 and mitochondrial SLC25A15 as mediators of adequate serine supply to sustain cancer cell proliferation.

## Main

Amino acids are indispensable precursors for protein biosynthesis and essential nutrients of central carbon metabolism, redox homeostasis and biosynthesis of nucleotides, polyamines and certain lipids^1,2^. A considerable body of research over the last 10 years has illustrated the importance of serine as a key nutrient supporting cancer cell metabolism, tumor growth and treatment resistance^3–14^. While cells can perform de novo serine synthesis, most transformed cells also rely on serine uptake to meet their increased biosynthetic requirements^15–17^. Serine is a major source of one-carbons entering the tetrahydrofolate (THF) cycle for nucleotide synthesis and is also critical for synthesis of proteins, antioxidants and certain lipids^16,18^. Overall, cellular serine availability is governed by two factors: uptake of extracellular serine and serine biosynthesis. Low de novo serine synthesis capability is a common occurrence in cancer^4,5,19^. Thus, limitation of exogenous serine, by restriction of dietary supply, is an effective treatment strategy in numerous preclinical models of cancer^3–11^; however, the key transporters responsible for the uptake of serine into cancer cells are unknown. SLCs, a family of transmembrane nutrient transport proteins, are the gatekeepers of amino acid acquisition and exchange in mammalian cells^20–22^ and are emerging as anticancer therapeutic targets^21,23^. Several previous studies using nonphysiological models have identified over ten SLC family members with the potential to transport serine (Supplementary Table 1), but functional studies in cancer cells under physiological conditions are lacking. A major potential obstacle with targeting serine transport as a therapeutic intervention is the possibility of transporter redundancy. A major challenge of the present study was therefore to identify whether a limited subset of SLCs was active in serine transport (with potential for therapeutic targeting) or a broader set of SLCs resistant to targeted manipulation. For this reason, our initial experiments, using a combined short interfering RNA (siRNA)/liquid chromatography–mass spectrometry (LC–MS) metabolomics screen, involved targeting all SLC family members.

The SLCs, a family of approximately 380 transmembrane proteins, are the principal means of amino acid transport in mammalian cells with an estimated 20% of the SLC superfamily being primarily dedicated to amino acid transport^20,24^. Transport mechanisms are highly heterogeneous between different SLC members; while they are generally ATP-independent, they can involve intracellular/extracellular amino acid gradients, Na^+^ influx or exchange of K^+^, H^+^, Cl^−^ or OH^−^ ions to catalyze amino acid uptake against their concentration gradient or even amino acid exchange^21^. SLCs demonstrate a remarkable substrate flexibility, with many SLCs being linked to the transport of a variety of amino acids and having ambiguous biological functions. For other SLCs, their exact substrates have not been fully characterized and many members of the SLC superfamily are currently deemed orphan, without known ligands^24^. Most of our current knowledge of SLC function is derived from biochemical studies, which lack cellular and pathophysiological context^25,26^. Considering that nutrient uptake is upregulated in cancer^21^, we sought to explore the involvement of the SLC superfamily in amino acid transport using assays providing functional information on substrate identity as well as phenotypic assessment of cell proliferation.

First, we applied an unbiased arrayed (one SLC-encoding gene targeted per well) RNA interference (RNAi) screen combining high-throughput imaging to assess cancer cell proliferation and LC–MS metabolomics (performed on each well) to map amino acid consumption. Using this functional amino acid consumption atlas, we identified SLC members involved in serine uptake. By combining quantitative stable-isotope tracing with functional cell growth assays, we aimed to identify SLC transporters expressed in cancer that directly or indirectly facilitate the uptake of extracellular serine to support cancer cell survival and proliferation in vitro and in vivo.

## Results

### Amino acid consumption atlas for SLCs

**Fig. 1:**
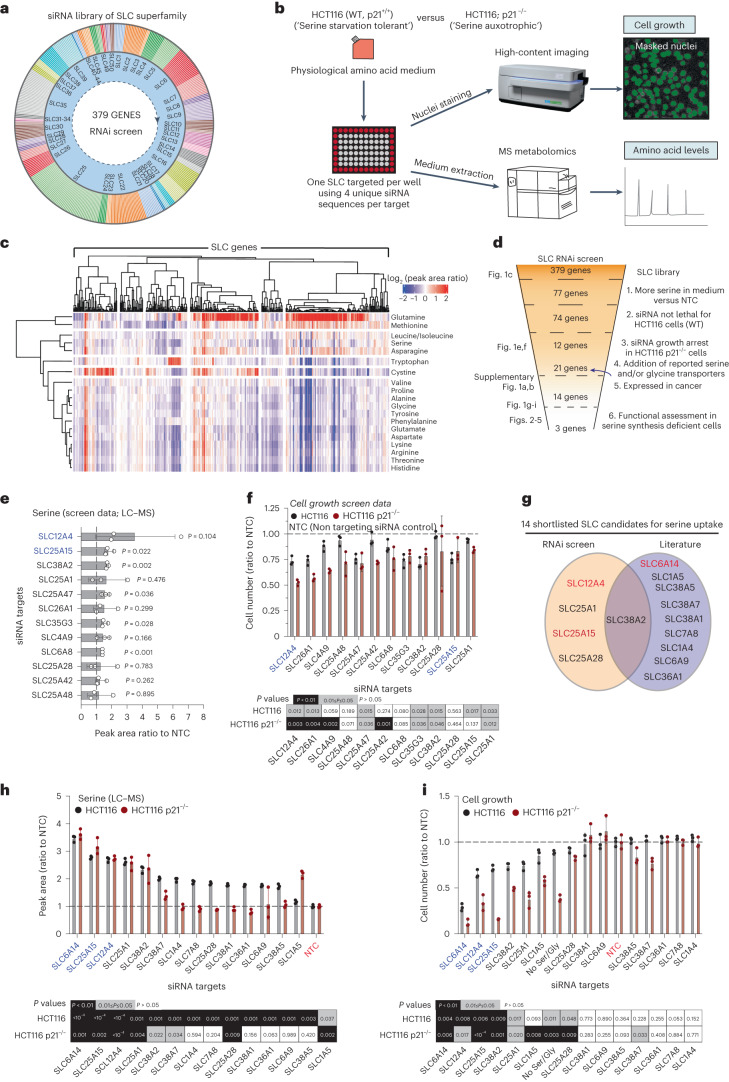
Phenotypic landscape of SLC superfamily reveals SLCs influencing serine uptake in cancer cells. **a**, Schematic of SLC RNAi library. **b**, Schematic of high-throughput imaging/LC-MS metabolomics strategy. WT, wild-type. **c**, Heat map showing amino acid usage of HCT116 cells upon knockdown of SLC-encoding genes. Values are log_2_ (peak area ratio to NTC). Heat map columns and rows are clustered based on Pearson’s correlation distance method. **d**, Strategy for hit selection and shortlisting. **e**, Serine levels in medium of HCT116 cells upon knockdown of SLC-encoding genes. Values are mean ± s.d. of peak area ratio to NTC and adjusted for cell number. **f**, Cell number (ratio to NTC) of HCT116 and HCT116 p21^−^^/^^−^ cells upon knockdown of SLC-encoding genes after 72 h of growth. Values are mean ± s.d. Heat map indicating *P* values for each condition compared to NTC (control) (bottom). **g**, Plot showing serine uptake SLC candidates selected for further investigation. **h**, Serine levels in medium of HCT116 and HCT116 p21^−^^/^^−^ cells upon knockdown of SLC-encoding genes using a deconvoluted siRNA for each gene. Values are peak area ratio to NTC and adjusted for cell number. Bar plots show mean ± s.d. Heat map indicating *P* values for each condition compared to NTC (control) (bottom). **i**, Cell number (ratio to NTC) of HCT116 and HCT116 p21^−^^/^^−^ cells upon knockdown of SLC-encoding genes using a deconvoluted siRNA for each gene, after 72 h of growth. Values are mean ± s.d. Heat map indicating *P* values for each condition compared to NTC (control) (bottom). Data are *n* = 3 biological replicates from individual experiments (**c**,**e**,**f**,**h**,**i**). Statistical significance was assessed by two-tailed one-sample *t*-test on natural log-transformed values (**e**,**f**,**h**,**i**). Source data

As SLC function can be regulated by nutrient concentration gradients^26^, we performed experiments in a formulated medium containing all proteinogenic amino acids at physiological concentrations. Using an arrayed siRNA library (one SLC targeted per well using a pool of four siRNA sequences), we silenced 379 genes from 50 families of the SLC superfamily in HCT116 cells (Fig. 1a,b). Following SLC gene silencing (72 h) we used high-content imaging to assess changes in cell number and sampled medium for LC–MS analyses of amino acid levels (Fig. 1c). Our approach revealed remarkably a broad involvement of SLCs in influencing, either directly or indirectly, consumption and release of amino acids. Specifically, silencing of ~85% of SLC-encoding genes seemed to alter the levels of at least one amino acid in the medium (Fig. 1c). Considering all SLC–amino acid combinations (all data points), most gene silencing events had little impact on amino acid abundance (change <20%) in the medium (52% of data points, white/pale colors in Fig. 1c). Silencing of SLC-encoding genes seemed in some cases to increase amino acid levels in the medium by more than 20%, suggesting decreased uptake (red colors, 16% of data points), whereas in approximately one third of cases it decreased them by more than 20%, indicating increased uptake (blue colors, 32% of data points) (Fig. 1c).

Silencing certain clusters of SLCs seemed to globally reduce or increase the uptake of most amino acids. Other clusters had opposing effects on different amino acids. This variability is not surprising and can be attributed to several factors. SLC proteins are not generally exclusive to one substrate and moreover, many of them are amino acid exchangers (where uptake of amino acids is coupled to the release of others) maintaining equilibrium of the total amino acid pool^24^. In the absence of one SLC, this can also be achieved through activation of other SLCs. Thus, when uptake of one amino acid is decreased (due to SLC knockdown), cells may respond by upregulating consumption of amino acids via different SLCs.

We also observed that silencing of several SLC genes had a substantial impact on glutamine uptake. Glutamine tends to be the most highly consumed amino acid by cancer cells^27,28^. It is notable in our dataset that when glutamine uptake is decreased, the uptake of other amino acids increases (Fig. 1c), perhaps through several different SLCs, as a regulatory mechanism of amino acid pool homeostasis. While interpreting this dataset, it is important to consider that amino acid consumption can be influenced by indirect events such as cellular demands for certain amino acids, differential activation of amino acid synthesis versus uptake and effects of SLC-mediated transport of other substrates (for example carbohydrates, ions or vitamins) that influence cellular amino acid metabolism. Thus, further characterization is necessary to functionally link SLCs to uptake of amino acids of interest.

### Identification of SLCs involved in serine uptake

**Extended Data Fig. 1:**
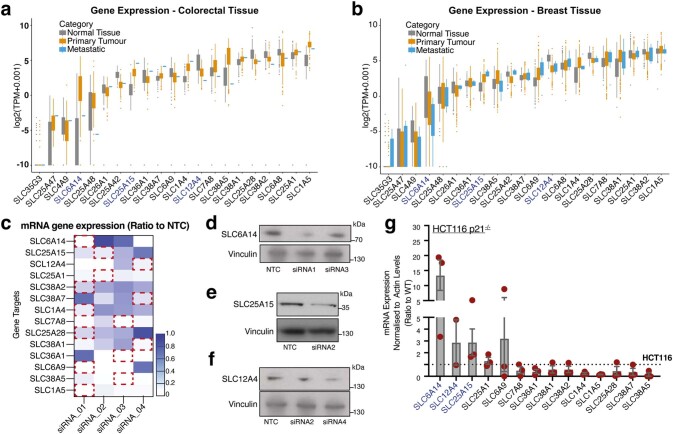
Phenotypic landscape of SLC superfamily reveals SLCs influencing serine transport in cancer cells. **a**. Gene expression levels of selected SLC-encoding genes in human colorectal tissues. Values are log2(TPM + 0.001) from n = 359 normal tissue, n = 380 primary tumor and n = 1 metastatic samples. **b**. Gene expression levels of selected SLC-encoding genes in human breast tissues. Values are log2(TPM + 0.001) from n = 292 normal tissue, n = 1092 primary tumor and n = 7 metastatic samples. **c**. Heat map showing gene silencing efficiency of 4 deconvoluted siRNAs against each SLC-encoding gene. Values are mRNA expression normalized to *ACTIN* levels and relative to NTC control. Red boxes indicate selected siRNA for further experiments. **d**. Immunoblots of SLC6A14 and Vinculin (loading control) in control (NTC) or SLC6A14-silenced HCT116 cells. Blots are representative of three independent experiments. **e**. Immunoblots of SLC25A15 and Vinculin (loading control) in control (NTC) or SLC25A15-silenced HCT116 cells. Blots are representative of three independent experiments. **f**. Immunoblots of SLC12A4 and Vinculin (loading control) in control (NTC) or SLC12A4-silenced HCT116 cells. Blots are representative of three independent experiments. **g**. mRNA expression levels of indicated SLC-encoding genes in HCT116 p21^-/-^ cells versus HCT116 WT control. Values are mean±s.e.m. from n = 3 independent experiments. Data in **a**,**b**:: Box and whiskers plots are log2 (TPM + 0.001) with box limits representing 25^th^ and 75^th^ percentiles of the dataset. Whiskers extend from the hinge to the largest or smallest value, corresponding to the upper or lower 1.5x IQR (inter-quartile range). Individual dot points represent data beyond the end of the whiskers.

To identify SLCs involved in serine uptake we applied several sequential filters to the screen data (Fig. 1d). First, we long-listed SLCs that when knocked down, decreased serine uptake from the medium (Fig. 1e). This long-list is predicted to contain two groups; SLCs that facilitate serine uptake and SLCs that do not facilitate serine uptake, but cause decreased proliferation when silenced, decreasing serine uptake as a side effect. To remove this confounding factor, we further shortlisted SLCs that had greater impact in serine-auxotrophic cells versus serine starvation-tolerant cells (Fig. 1f); HCT116 cells tolerate serine starvation with an approximately 50% decrease in proliferation, whereas HCT116 p21^−^^/^^−^ cells are serine auxotrophs^8^. p21 is an essential mediator of cell survival upon serine starvation, through cell cycle arrest and inhibition of nucleotide synthesis. Thus, absence of extracellular serine for p21-depleted cells leads to cell death^8^. While there is a lack of previous data attributing specific SLCs to serine transport in cancer, several SLCs have been linked to serine transport in nonpathological models or other disease types^24^. Therefore, we also incorporated into the shortlist plasma membrane-localized SLCs previously reported to mediate serine uptake (Supplementary Table 1). To ensure relevance to cancer, we next asked whether shortlisted genes were expressed in colorectal and breast cancer; two major tumor types in which serine metabolism has been studied^8,9,29^ (Extended Data Fig. 1a,b), which narrowed our list to 14 SLC-encoding genes that could be involved in serine transport and have relevance to cancer (Fig. 1g).

For the 14 shortlisted SLC genes, we deconvoluted the siRNA pools to select single siRNAs with effective silencing (Extended Data Fig. 1c). As siRNA pools (four RNA oligonucleotides) may produce milder silencing effects than single highly potent oligonucleotides, we selected for each gene of interest the optimal siRNA that conferred the greatest reduction in gene expression. Even with these optimal siRNAs there is still some residual protein expression as evidenced in the case of SLC6A14, SLC25A15 and SLC12A4 (Extended Data Fig. 1d–f). Using high-content imaging and LC–MS we confirmed to what degree single siRNA-mediated silencing of our shortlisted genes impacted serine uptake and proliferation, finding that SLC6A14, SLC25A15 and SLC12A4 had the greatest impact (Fig. 1h,i). Given their serine auxotrophy, we hypothesized that HCT116 p21^−^^/^^−^ cells would have higher basal expression of SLCs mediating serine uptake versus HCT116 and found that, similar to cell growth and metabolomics assays, *SLC6A14*, *SLC12A4* and *SLC25A15* stood out, displaying 3–15-fold increased expression (Extended Data Fig. 1g).

### Serine synthesis deficiency enhances SLC activity

**Fig. 2:**
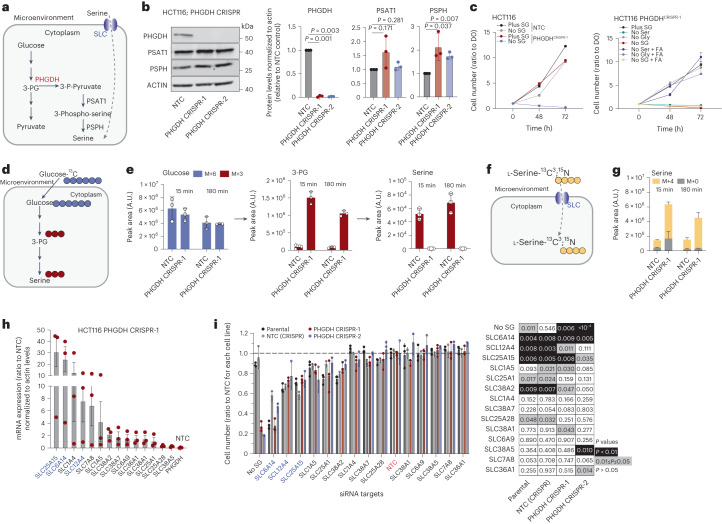
Serine synthesis-deficient cells depend on SLC-mediated serine uptake. **a**, Schematic of serine synthesis pathway. **b**, Immunoblots of PHGDH, PSAT1, PSPH and ACTIN (loading control) in control (NTC) or PHGDH-depleted HCT116 cells (left). Blots are representative of three independent experiments. Densitometric quantification of protein (right). Values are mean ± s.d. from *n* = 3 independent experiments. Statistical significance assessed by two-tailed one-sample *t*-test on natural log-transformed values. **c**, Growth curves of HCT116 NTC (control) or PHGDH-depleted cells in medium ± SG (left). SG, serine and glycine. Growth curves of HCT116 PHGDH-depleted cells in medium ± serine and/or glycine ± formic acid (FA) (right). Values are mean ± s.e.m. from *n* = 3 biological replicates and relative to *t* = 0 (D0). **d**, Schematic of [U-^13^C_6_]d-glucose administration to monitor serine synthesis. **e**, Incorporation of glucose-derived, labeled carbons into 3-phosphoglycerate (3-PG) and serine in HCT116 NTC (control) or PHGDH-depleted cells following 15 min and 180 min incubation in medium supplemented with [U-^13^C_6_]d-glucose. Values are mean ± s.d. from *n* = 3 biological replicates. **f**, Schematic of [^13^C_3_,^15^N]l-serine administration to monitor serine uptake. **g**, Serine levels in HCT116 NTC (control) or PHGDH-depleted cells showing [^13^C_3_,^15^N]l-serine-derived labeled carbon and nitrogen incorporation following 15 min and 180 min incubation in medium supplemented with [^13^C_3_,^15^N]l-serine. Values are mean ± s.d. from *n* = 3 biological replicates. **h**, Messenger RNA expression levels of indicated SLC-encoding genes in HCT116 PHGDH-depleted cells versus HCT116 NTC control. Values are mean ± s.e.m. from *n* = 3 independent experiments. **i**, Cell number (ratio to NTC) of HCT116 parental, NTC (CRISPR control) and two PHGDH-depleted cell lines upon knockdown of indicated SLC-encoding genes, after 72 h of growth (left). Values are mean ± s.d. from *n* = 3 biological replicates. Heat map indicating *P* values for each condition compared to NTC (control) (right). Statistical significance was assessed by two-tailed one-sample *t*-test on natural log-transformed values. Source data

**Extended Data Fig. 2:**
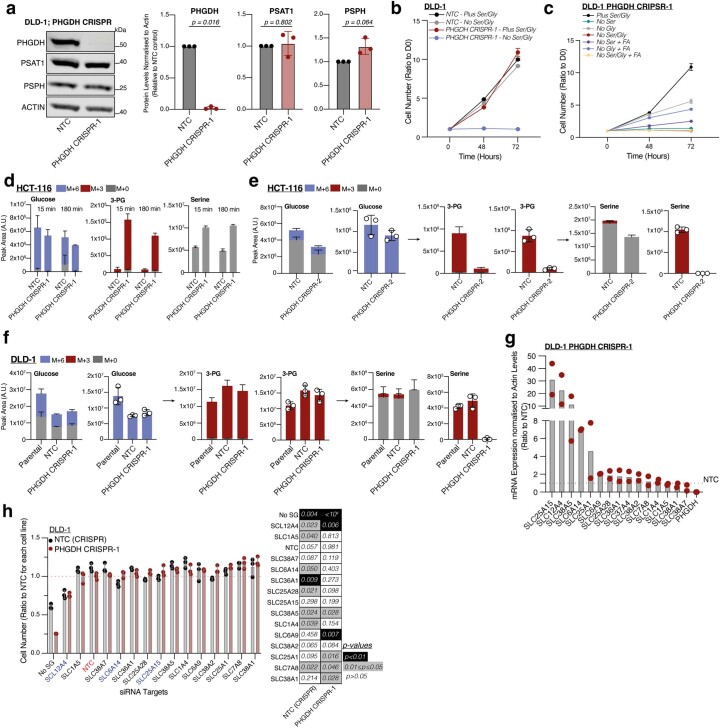
Serine synthesis-deficient cells depend on SLC-mediated serine uptake. **a. Left**; Immunoblots of PHGDH, PSAT1, PSPH and ACTIN (loading control) in control (NTC) or PHGDH-depleted DLD-1 cells. Blots are representative of three independent experiments. **Right**; densitometric quantification of protein. Values are mean±s.d. from n = 3 independent experiments. Statistical significance assessed with two-tailed one-sample *t*-test on natural log-transformed values. **b**. Growth curves of DLD-1 NTC (control) or PHGDH-depleted cells in media ± serine and glycine. Values are mean±s.e.m. from n = 3 biological replicates and relative to t = 0. **c**. Growth curves of DLD-1 PHGDH-depleted cells in media ± serine and/or glycine ± formic acid (FA). Values are mean±s.e.m. from n = 3 biological replicates and relative to t = 0. **d-e**. Incorporation of glucose-derived, labeled carbons into 3-phosphoglycerate (3-PG) and serine in HCT116 NTC (control) or PHGDH CRISPR-1 **(d)** and PHGDH CRISPR-2 **(e)** depleted cells following 15 min and 180 min incubation in media supplemented with [U-^13^C_6_]glucose. Values are mean±s.d. from n = 3 biological replicates. **f**. Incorporation of glucose-derived, labeled carbons into 3-phosphoglycerate (3-PG) and serine in DLD-1 NTC (control) or PHGDH-depleted cells following 15 min and 180 min incubation in media supplemented with [U-^13^C_6_]glucose. Values are mean±s.d. from n = 3 biological replicates. **g**. mRNA expression levels of indicated SLC genes in DLD-1 PHGDH-depleted cells versus DLD-1 NTC control. Values are mean±s.d. from n = 2 independent experiments. **h. Left;** Cell number (Ratio to NTC) of DLD-1 NTC (CRISPR control) and PHGDH-depleted cells upon knockdown of indicated SLC, after 72 hours of growth. Values are mean±s.d. from n = 3 biological replicates. No SG; no Serine/Glycine condition. **Right**; Heat map indicating *p*-values for each condition compared to NTC (siRNA control). Statistical significance was assessed by two-tailed one-sample *t*-test on natural log-transformed values.

Serine can be biosynthesized de novo from the glycolytic intermediate 3-phosphoglycerate (3-PG) through reactions catalyzed by phosphoglycerate dehydrogenase (PHGDH), phosphoserine aminotransferase (PSAT1) and phosphoserine phosphatase (PSPH)^17^. While de novo serine synthesis is upregulated in some tumor types^29^, most tumors rely on some degree of exogenous serine uptake, with many displaying low serine synthesis capability^4,5,19^. For tumors relying on serine uptake, dietary restriction of serine is detrimental to tumor growth^3–11^. For tumors able to effectively perform de novo serine synthesis a combination of dietary serine restriction and serine synthesis inhibition is needed to limit tumor growth^11^. We hypothesized that cancer cells with low de novo serine synthesis would be more dependent on SLC-mediated serine uptake. Using CRISPR-Cas9 we deleted *PHGDH* (Fig. 2a) in HCT116 and DLD-1 colorectal cancer cells (Fig. 2b and Extended Data Fig. 2a). While *PHGDH*-deleted cells proliferated at similar rates to non-targeting control (NTC) cells in complete medium, as expected, their growth was inhibited by serine and glycine starvation (Fig. 2c and Extended Data Fig. 2b). Supplementation with the one-carbon donor formic acid and glycine rescued HCT116 cells fully, and DLD-1 cells partially, from serine starvation, highlighting the importance of serine-dependent one-carbon metabolism in these cells (Fig. 2c and Extended Data Fig. 2c). To confirm loss of serine synthesis upon *PHGDH* deletion, we performed tracing experiments with [U-^13^C_6_]-d-glucose (Fig. 2d). Lack of serine synthesis was confirmed in two *PHGDH*-deleted HCT116 cell lines (Fig. 2e and Extended Data Fig. 2d,e) and *PHGDH*-deleted DLD-1 cells (Extended Data Fig. 2f).

We next assessed whether PHGDH loss promoted serine uptake using [^13^C_3_,^15^N]l-serine (Fig. 2f). Cells lacking PHGDH displayed markedly higher intracellular [^13^C_3_,^15^N]l-serine levels compared to NTC controls (Fig. 2g), suggesting strongly enhanced serine uptake. Considering the essentiality of serine uptake for proliferation upon loss of PHGDH, we assessed whether this could be mediated through increased expression of our shortlisted SLC candidates. Under fully fed conditions *PHGDH*-deleted cells showed increased expression of shortlisted SLCs; in similarity to the data from serine-auxotrophic HCT116 p21^−^^/^^−^ cells, *SLC25A15*, *SLC12A4* and *SLC6A14* were among the most elevated in terms of gene expression in *PHGDH*-deleted cells (Fig. 2h and Extended Data Fig. 2g).

Given the simultaneous expression of multiple serine transport-related SLCs in these cells, we assessed whether silencing individual SLCs would be adequate to decrease proliferation in *PHGDH*-deleted cells or whether SLC redundancy could overcome loss of a single SLC. We acutely silenced 14 shortlisted SLCs in parental, NTC and two *PHGDH*-deleted HCT116 cell lines. After 72 h we observed that *SLC6A14*, *SLC12A4*, *SLC25A15* and *SLC38A2* silencing decreased proliferation in all four cell lines, but without a greater impact on *PHGDH*-deleted cells (Fig. 2i). DLD-1 cells were more resilient to RNAi silencing of the shortlisted SLCs, among them, *SLC12A4* silencing caused a similar growth inhibition in NTC (CRISPR control) and *PHGDH*-deleted cells (Extended Data Fig. 2h). Thus, cells were generally able to tolerate, with varying degrees of growth inhibition, the loss of single SLCs involved in serine transport, even when unable to biosynthesize serine de novo, suggesting compensatory serine uptake by other SLCs (some degree of transporter redundancy).

### SLC6A14, SLC38A2 and SLC12A4 influence serine uptake

Based on these experiments, we narrowed the initial list of 14 genes to 8 SLCs; *SLC6A14*, *SLC12A4*, *SLC25A15*, *SLC38A2*, *SLC38A1*, *SLC38A5*, *SLC1A5* and *SLC1A4*. It has been reported that SLC6A14, SLC38A2, SLC38A1, SLC38A5, SLC1A5 and SLC1A4 are plasma membrane amino acid transporters^24^, whereas SLC25A15 is a member of the mitochondrially localized SLC25 family and has been previously linked to ornithine transport^24,30^. SLC12A4 is a putative plasma membrane potassium (K^+^) and chloride (Cl^−^) ion exhanger^31^. To directly establish whether these eight SLCs were involved in serine uptake, we developed two assays, one measuring [^13^C_3_,^15^N]l-serine consumption rates from the medium over 8 h and one monitoring intracellular serine dynamics on acute exposure to [^13^C_3_,^15^N]l-serine. For these assays we employed a combination of RNAi silencing of SLCs in HCT116 cells and SLC overexpression in HEK293 (transient) and/or HCT116 (stable) cells.

**Fig. 3:**
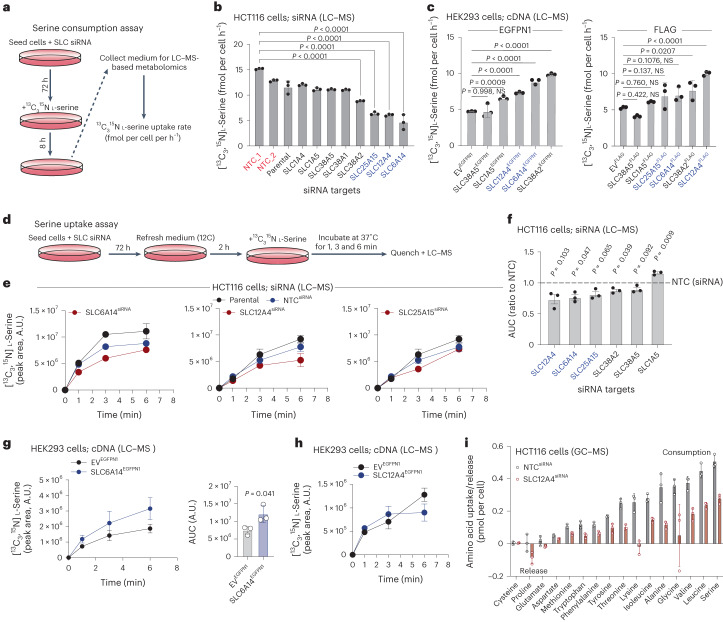
Functional characterization of shortlisted SLCs in serine uptake. **a**, Schematic of [^13^C_3_,^15^N]l-serine consumption assay. **b**, The [^13^C_3_,^15^N]l-serine consumption in HCT116 cells silenced for the indicated SLC-encoding genes. **c**, The [^13^C_3_,^15^N]l-serine consumption in HEK293 cells overexpressing indicated GFP- (left) or FLAG-tagged (right) SLC-encoding genes. NS, not significant. **d**, Schematic of [^13^C_3_,^15^N]l-serine uptake assay. **e**, The [^13^C_3_,^15^N]l-serine uptake over time of HCT116 parental, NTC (siRNA control) and HCT116 cells silenced for SLC6A14, SLC12A4 or SLC25A15 as indicated. Values are mean ± s.e.m. from *n* = 3 biological replicates. **f**, Area under the curve (AUC) of [^13^C_3_,^15^N]l-serine uptake curves as on **e** of HCT116 cells silenced for indicated SLCs. **g**, The [^13^C_3_,^15^N]l-serine uptake of HEK293 EV^EGFPN1^ (control) or SLC6A14^EGFPN1^ overexpressing cells (left). Values are mean ± s.d. from *n* = 3 biological replicates (left). AUC of [^13^C_3_,^15^N]l-serine uptake curves from cells on left (right). Values are mean ± s.d. from *n* = 3 biological replicates. Statistical significance was assessed with two-tailed unpaired *t*-test. **h**, The [^13^C_3_,^15^N]l-serine uptake of HEK293 EV^EGFPN1^ (control) and SLC12A4^EGFPN1^ cells. **i**, Amino acid uptake or release levels (pmol per cell) over 24 h of HCT116 cells NTC (siRNA control) or silenced for SLC12A4. Statistical significance was assessed with ordinary one-way analysis of variance (ANOVA) and Dunnett’s multiple comparisons test (**b**,**c**,**f**). Data values are mean ± s.d. from *n* = 3 biological replicates (**b**,**c**,**f**,**h**,**i**).

**Extended Data Fig. 3:**
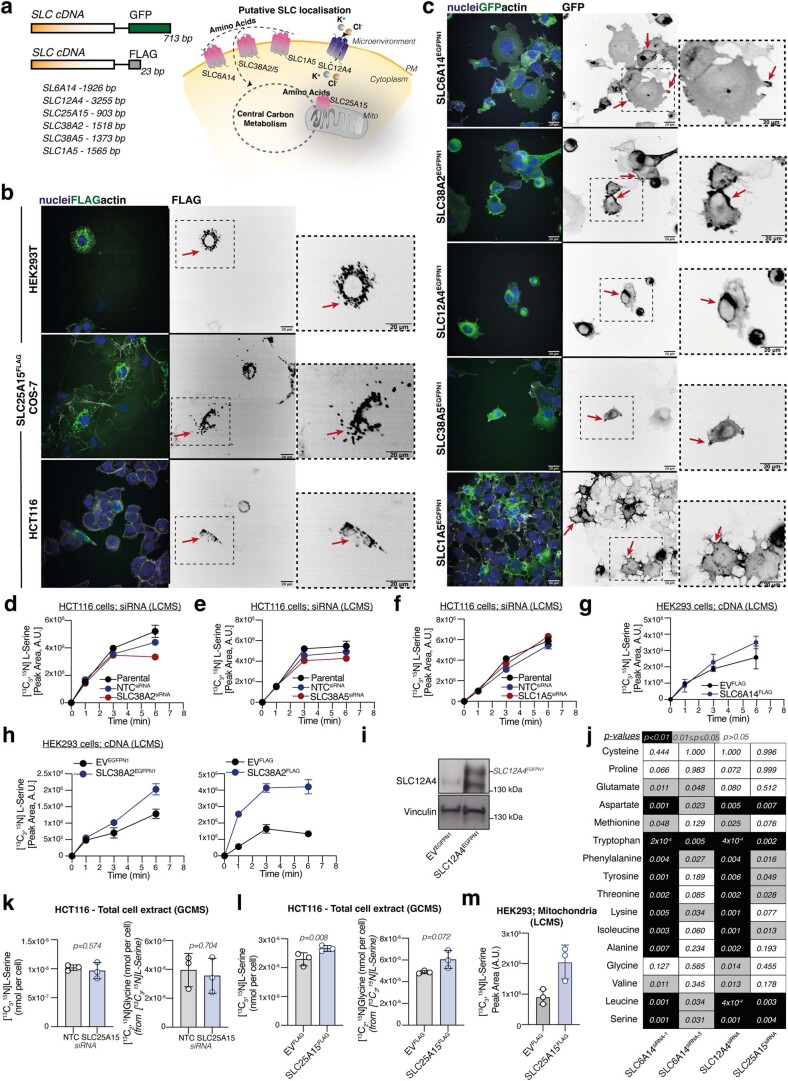
Functional identification of shortlisted SLCs in serine uptake. **a. Left;** Schematic of SLC tagging for overexpression studies. **Right;** Schematic of observed and reported localization/functionality of shortlisted SLCs. **b. Left**; Immunofluorescence of HEK293 (top), COS7 (middle) and HCT116 (bottom) cells expressing SLC25A15 tagged with FLAG showing nuclei (blue), anti-FLAG (green) and actin (gray). Middle; anti-FLAG staining (gray). **Right**; Magnification of areas indicated by black boxes. Red arrows indicate representative localization. Scale bars; 20 μm. Images are representative from n = 3 biological replicates. **c. Left**; Immunofluorescence of HEK293 cells expressing the indicated SLC proteins tagged with GFP showing nuclei (blue), anti-GFP (green) and actin (gray). Middle; anti-GFP staining (gray). **Right**; Magnification of areas indicated by black boxes. Red arrows indicate representative localization for each SLC protein. Scale bars; 20 μm. Images are representative from n = 3 biological replicates. **d-f**. [^13^C_3_,^15^N]L-Serine uptake of HCT116 Parental, NTC siRNA (control) and cells silenced for SLC38A5 **(d)**, SLC1A5 **(e)** or SLC25A15 **(f)**. Values are mean±s.e.m. from n = 3 biological replicates. **g**. [^13^C_3_,^15^N]L-Serine uptake of HEK293 EV^FLAG^ (control) or SLC6A14^FLAG^ overexpressing cells. Values are mean±s.d. from n = 3 biological replicates. **h**. [^13^C_3_,^15^N]L-Serine uptake of HEK293 EV^EGFPN1^ (control) and SLC38A2 ^EGFPN1^ (**left**) or EV^FLAG^ (control) or SLC38A2^FLAG^ (**right**) cells. Values are mean±s.e.m. from n = 3 biological replicates. **i**. Immunoblots of SLC12A4 and Vinculin (loading control) in HCT116 cells expressing GFP (EV^EGFPN1^) or GFP-tagged SLC12A4. Blots are representative of three independent experiments. **j**. Heat map indicating *p*-values of amino acid uptake comparison between SLC6A14, SLC12A4, SLC25A15 to NTC (siRNA control) in HCT116 cells. Data linked to Figs. 3i, 4h and 5a. **k**. [^13^C_3_,^15^N]L-Serine levels (nmol per cell) (**left**) and [^13^C_2_,^15^N]Glycine levels (nmol per cell) (**right**) of HCT116 NTC (siRNA control) and HCT116 SLC25A15-silenced cells. Statistical significance was assessed with two-tailed unpaired *t*-test. **l**. [^13^C_3_,^15^N]L-Serine levels (nmol per cell) (**left**) and [^13^C_2_,^15^N]Glycine levels (nmol per cell) (**right**) of HCT116 FLAG (EV^FLAG^, control) and HCT116 FLAG-tagged SLC25A15-overexpressing cells. Statistical significance was assessed with two-tailed unpaired *t*-test. **m**. [^13^C_3_,^15^N]L-Serine uptake in mitochondria-enriched fractions of HEK293 cells overexpressing EV^FLAG^ or SLC25A15^FLAG^ and supplemented with [^13^C_3_,^15^N]L-Serine for 60 min. Data in k-m are mean±s.d. from n = 3 biological replicates.

Initially, we performed serine consumption assays using [^13^C_3_,^15^N]l-serine over 8 h (Fig. 3a). Specifically, we measured extracellular [^13^C_3_,^15^N]l-serine levels in the culture medium, as a function of serine uptake and release, providing a net readout of serine uptake (fmol per cell h^−1^). While cells may not take up serine at a constant rate for the entirety of this period, shorter term uptake assays suggest that uptake will be at steady state for the vast majority of this time. Acute silencing of *SLC6A14*, *SLC12A4*, *SLC25A15* and *SLC38A2* in HCT116 cells decreased serine consumption, whereas *SLC1A4*, *SLC1A5*, *SLC38A1* and *SLC38A5* silencing had minimal impact (Fig. 3a,b). We therefore shortlisted the top four SLCs (SLC6A14, SLC12A4, SLC25A15 and SLC38A2) and included SLC1A5 and SLC38A5 as controls for further experiments. We overexpressed the selected SLCs using complementary DNA tagged with green fluorescent protein (GFP) or FLAG (Extended Data Fig. 3a) in human embryonic kidney (HEK293) cells, which have been previously used in transporter overexpression studies^32,33^ and assessed subcellular localization and serine consumption. For most SLCs both GFP- and FLAG-tagged versions were functional, as assessed by localization, expression levels and impact on amino acid transport, whereas for SLC25A15 only the FLAG tag version seemed functional. SLC6A14, SLC12A4, SLC38A2, SLC38A5 and SLC1A5 localized to the plasma membrane, whereas SLC25A15 localized to mitochondria (Extended Data Fig. 3a–c). Overexpression of SLC38A2, SLC6A14 and SLC12A4 (but not of SLC1A5, SLC25A15 and SLC38A5) increased serine consumption rates in HEK293 cells (Fig. 3c). To assess serine uptake dynamics, we analyzed intracellular accumulation of [^13^C_3_,^15^N]l-serine following acute SLC gene silencing (Fig. 3d). We observed that *SLC6A14*, *SLC12A4* and *SLC25A15*, but not *SLC38A2*, *SLC38A5* or *SLC1A5* silencing decreased serine uptake (Fig. 3e,f and Extended Data Fig. 3d–f).

In HEK293 cells we observed that overexpression of SLC6A14 or SLC38A2 increased serine uptake (Fig. 3g and Extended Data Fig. 3g,h). Regarding SLC38A2, this may suggest that it is a plasma membrane-localized amino acid transporter capable of serine transport; however, as SLC38A2 silencing in HCT116 cells did not decrease serine uptake, it may not be a major endogenous mediator of serine transport in colorectal cancer cells. In fact, SLC38A2 has been linked to alanine transport in pancreatic cancer cells^34^ and glutamine transport in breast cancer cells^35^.

While knockdown assays in HCT116 cells suggest that SLC12A4 promotes serine transport, overexpression in HEK293 cells did not further increase serine uptake (Fig. 3h and Extended Data Fig. 3i), suggesting its role may be more indirect. SLC12A4 (also known as KCC1) transports chloride and potassium ions and has been shown to have a housekeeping role in the regulation of cellular volume^36^. As many amino acid transporters, notably including SLC6A14, depend on co-transport of amino acids with chloride ions, these data suggest that SLC12A4 has an important role in facilitating amino acid transport. We employed gas chromatography (GC)–MS metabolomics to quantitatively measure amino acid amounts consumed by SLC12A4-silenced HCT116 cells over 24 h. Our data suggest a generalized decrease of amino acid uptake upon SLC12A4 silencing in these cells (Fig. 3i and Extended Data Fig. 3j). While this result suggests that SLC12A4 activity may generally facilitate amino acid import, the data may be confounded to some degree by the fact that impeding uptake of key amino acids like serine would slow proliferation and lower overall cellular amino acid demands.

### SLC25A15 is a mitochondrial serine transporter

**Fig. 4:**
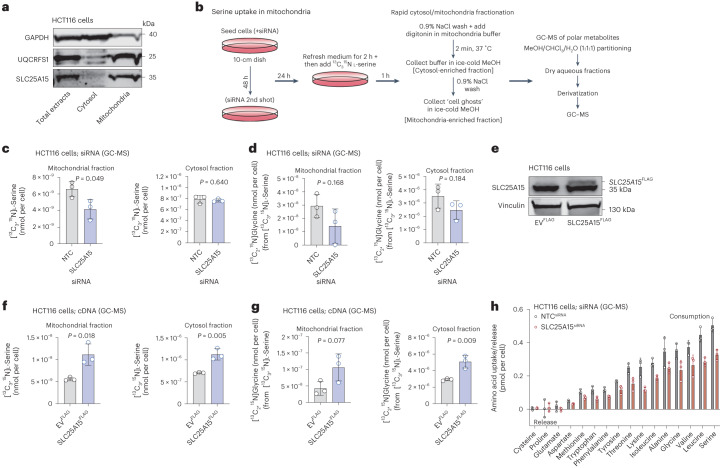
SLC25A15 is a mitochondrial serine transporter in colorectal cancer cells. **a**, Immunoblotting of GAPDH (cytosol marker), UQCRFS1 (mitochondrial marker) and SLC25A15 in whole, cytosol and mitochondrial lysate fractions from HCT116 cells. Blots are representative of three independent experiments. **b**, Schematic of [^13^C_3_,^15^N]l-serine uptake assay followed by cytosol/mitochondrial fractionation. **c**, The [^13^C_3_,^15^N]l-serine uptake (nmol per cell) of HCT116 NTC (siRNA control) and HCT116 SLC25A15-silenced cells in mitochondrial-enriched (left) and cytosol-enriched (right) fractions. **d**, The [^13^C_2_,^15^N]glycine levels (nmol per cell) in mitochondrial-enriched (left) and cytosol-enriched (right) fractions of cells from **c**. **e**, Immunoblotting of vinculin (loading control) and SLC25A15 in HCT116 cells expressing EV^FLAG^ (control) or FLAG-tagged SLC25A15. Blots are representative of three independent experiments. **f**, [^13^C_3_,^15^N]l-serine uptake (nmol per cell) in mitochondrial-enriched (left) and cytosol-enriched (right) fractions of HCT116 cells expressing EV^FLAG^ or FLAG-tagged SLC25A15. **g**, [^13^C_2_,^15^N]glycine levels (nmol per cell) in mitochondrial-enriched (left) and cytosol-enriched (right) fractions of cells from **f**. **h**, Amino acid uptake or release levels (pmol per cell) over 24 h of HCT116 cells NTC (siRNA control) or silenced for SLC25A15. Control data (NTC) from the same experiment are shown in Fig. 3i. Data values are mean ± s.d. from *n* = 3 biological replicates (**c**,**d**,**f**–**h**). Statistical significance was assessed with two-tailed unpaired *t*-test (**c**,**d**,**f**,**g**).

Members of the SLC25 family are mitochondrial localized transporters^37^. SLC25A15–FLAG localized to mitochondria in HEK293, Cos7 and HCT116 cells (Extended Data Fig. 3b). To assess the localization of endogenous SLC25A15, we fractionated HCT116 cells and detected SLC25A15 expression only in the mitochondrial fraction, similar to the mitochondrial marker UQCRFS1 (Fig. 4a). We next asked whether SLC25A15 is able to transport serine into mitochondria. Mitochondrial metabolism of serine, via SHMT2, is the major route of serine catabolism in cancer cells providing the majority of one-carbon units for biosynthetic purposes^38^. To test SLC25A15 involvement in mitochondrial serine transport we supplemented SLC25A15-silenced or NTC HCT116 cells with [^13^C_3_,^15^N]l-serine for 60 min. We then permeabilized cells with digitonin and rapidly isolated metabolites from cytosol-enriched and mitochondria-enriched fractions followed by GC–MS-based quantification of amino acids (Fig. 4b).

SLC25A15-silenced cells displayed less labeled serine in the mitochondrial but not in the cytosolic fractions, nor in total cell extracts (Fig. 4c and Extended Data Fig. 3k). This was associated with a trend for lower incorporation of serine-derived carbons and nitrogen into mitochondrial glycine, suggesting that SLC25A15 imports serine in mitochondria that is then converted into glycine (and formate) (Fig. 4d and Extended Data Fig. 3k). Next, we generated HCT116 cells stably expressing FLAG-tagged SLC25A15 (Fig. 4e) and subjected them to [^13^C_3_,^15^N]l-serine labeling followed by metabolite isolation from cytosol/mitochondrial fractions and GC–MS quantification. SLC25A15 overexpression in HCT116 cells increased labeled serine uptake by mitochondria, but also serine levels in the cytosol and in whole cell extracts (Fig. 4f and Extended Data Fig. 3l), suggesting that SLC25A15 overexpression promotes mitochondrial serine catabolism which increases serine requirements and influences overall cellular serine demands. Serine-derived labeled carbon and nitrogen incorporation into glycine were also higher following SLC25A15 overexpression (Fig. 4g and Extended Data Fig. 3l).

By performing mitochondrial compartmentalization followed by LC–MS metabolomics^39^ in HEK293 cells overexpressing FLAG or SLC25A15–FLAG and labeled with [^13^C_3_,^15^N]l-serine, we also observed that SLC25A15–FLAG expressing HEK293 cells displayed increased mitochondrial serine uptake (Extended Data Fig. 3m). SLC25A15 (also known as ORC1 and ORNT1) has also been previously shown to transport ornithine from the cytosol across the inner mitochondrial membrane to the mitochondrial matrix. Mutations in the gene encoding ORC1 have been linked to the hyperornithinemia-hyperammonemia-homocitrullinuria syndrome^40^. The properties of the human SLC25A15 have been initially studied mainly by overexpression in bacteria and reconstitution into liposomes. Fiermonte et al. reported that SLC25A15 is able to transport ornithine, lysine, arginine into the mitochondrial matrix, and exports citrulline from the matrix to the cytosol. In this previous study, serine transport was not studied as it was absent from the substrate buffer, but ORC1 seemed to have a preference for l-amino acids^41^. HCT116 cells silenced for SLC25A15 took up less serine and leucine with modest effects in other amino acids, as measured by GC–MS-based quantification of medium amino acids (Fig. 4h and Extended Data Fig. 3j). We conclude that this reflects the influence of mitochondrial SLC25A15 activity (which supports one-carbon metabolism and growth/proliferation) on global cellular nutrient demands, rather than a direct role in cellular import of a wide range of amino acids.

### SLC6A14 is a serine transporter in cancer cells

**Fig. 5:**
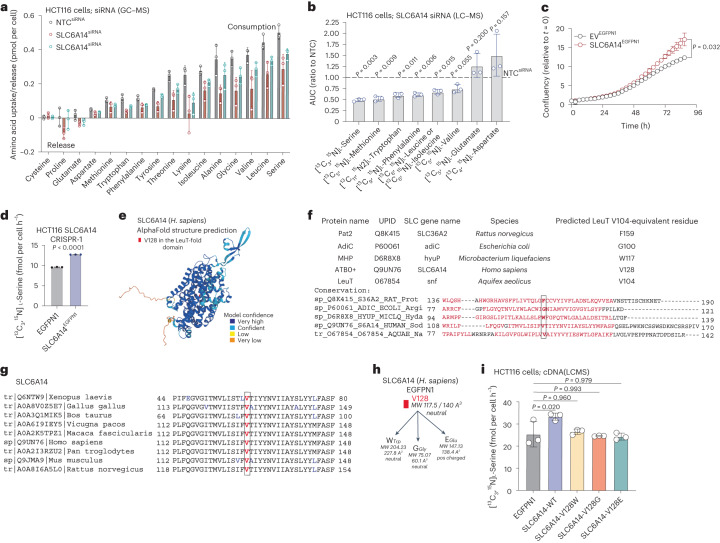
SLC6A14 LeuT domain mediates serine transport in colorectal cancer cells. **a**, Amino acid uptake or release levels (pmol per cell) over 24 h of HCT116 cells NTC (siRNA control) or silenced for SLC6A14. Control data (NTC) from the same experiment are also shown in Figs. 3i and 4h. **b**, Growth curves of HCT116 cells expressing EV^EGFPN1^ (empty vector, control) or GFP-tagged SLC6A14. Values are mean ± s.d. from *n* = 3 biological replicates and representative of three independent experiments. Statistical significance assessed by two-tailed Welch’s *t*-test at *t* = 87 h. **c**, AUC from uptake curves of SLC6A14-silenced HCT116 cells labeled with a pool of labeled amino acids as indicated for 1, 3 and 6 min. Values are mean ± s.d. and relative to NTC siRNA control from *n* = 3 biological replicates. Statistical significance was assessed with two-tailed one-sample *t-*test on natural log-transformed values. **d**, [^13^C_3_,^15^N]l-serine consumption in HCT116 SLC6A14-depleted cells overexpressing either EV^EGFPN1^ (EV, control) or SLC6A14^EGFPN1^. Values are mean ± s.d. from *n* = 3 biological replicates. Statistical significance assessed by two-tailed Welch’s *t*-test. **e**, Structure of the human SLC6A14 protein as predicted by AlphaFold2. **f**, Sequence alignment of different LeuT-containing transporters to predict equivalent residues to V104 of the LeuT transporter. **g**, Sequence alignment of SLC6A14 protein between different organisms. **h**, Cartoon specifying mutagenesis strategy on SLC6A14 protein. **i**, [^13^C_3_,^15^N]l-serine consumption in HCT116 cells overexpressing EGFPN1 (EV control) or GFP-tagged SLC6A14 WT, -V128W, -V128G and -V128E. Values are mean ± s.d. from *n* = 3 biological replicates. Statistical significance was assessed using ordinary ANOVA and Dunnett’s multiple comparisons test.

SLC6A14 (also known as ATB0^+^) is reported as a plasma membrane Na^+^/Cl^−^-dependent transporter (co-transport of two Na^+^, one Cl^−^ and one amino acid molecule) of a range of amino acids, upregulated in cancer^42–46^ for which serine is a theoretical substrate^20,47^. Functional characterization of human SLC6A14 in *Xenopus* oocytes using electrophysiology and radiolabeled amino acid uptake experiments, suggested that SLC6A14 can transport 18 of the 20 proteinogenic amino acids (with aspartate and glutamate the exceptions). In this previous study, six amino acids (isoleucine, leucine, methionine, valine, phenylalanine and tryptophan) had lower half-maximum effective concentration (EC_50_) values (more facile uptake) than serine (EC_50_ = 45 ± 5 μM)^48^; however, serine was not followed up with direct tracing analysis and is very likely that substrate affinity of SLCs can be influenced not only by patho/physiological conditions and cell line or tissue of origin examined, but also by the concentration balance between the different substrates in the medium or buffer of the study. GC–MS metabolomics quantification of amino acids in the medium of HCT116 cells silenced for SLC6A14, suggests that the uptake of serine is predominantly impeded, while the uptake of other amino acids such as leucine, glycine and lysine is also affected (Fig. 5a and Extended Data Fig. 3j). Notwithstanding the potential confounding factor of reduced proliferation, these results show that SLC6A14 transports a range of amino acids, with serine a dominant substrate in cancer cells.

**Extended Data Fig. 4:**
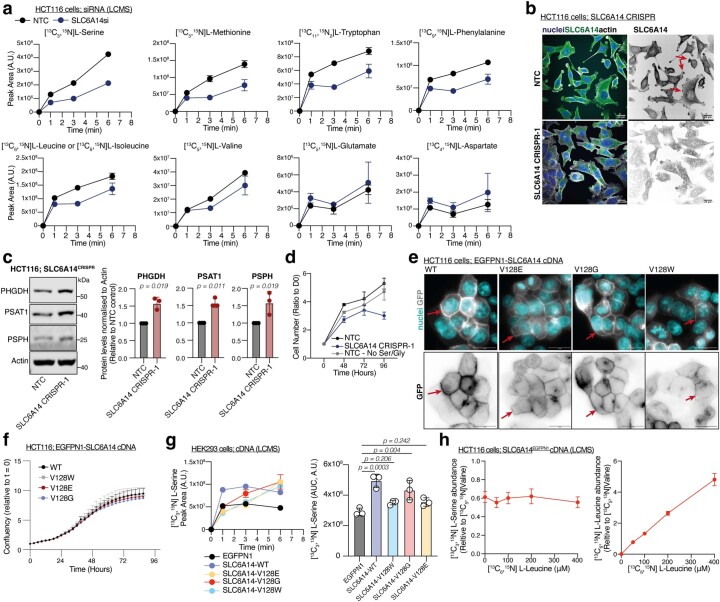
Functional identification of SLC6A14 in serine uptake. **a**. Uptake curves SLC6A14-silenced HCT116 cells supplemented with a pool of the indicated labeled amino acids for -1, -3 and -6 min. Values are mean±s.d. from n = 3 biological replicates. **b. Left**; Immunofluorescence of control (NTC) or SLC6A14-depleted HCT116 cells expressing showing nuclei (blue), anti-SLC6A14 (green) and actin (gray). **Right**; anti-SLC6A14 staining (gray). Red arrows indicate representative localization of SLC6A14. Scale bars; 20 μm. Images are representative from n = 3 biological replicates. **c. Left**; Immunoblots of PHGDH, PSAT1, PSPH and ACTIN (loading control) in control (NTC) or SLC6A14-depleted HCT116 cells. Blots are representative of three independent experiments. **Right**; densitometric quantification of protein. Values are mean±s.d. from n = 3 independent experiments. Statistical significance assessed with two-tailed one-sample *t*-test on natural log-transformed values. **d**. Growth curves of HCT116 NTC (CRISPR control) or SLC6A14-depleted cells. Values are mean±s.d. from n = 3 biological replicates. No Ser/Gly – No Serine/Glycine condition. **e. Top;** Immunofluorescence of HCT116 cells expressing GFP-tagged WT SLC6A14 or V128E, V128G and V128W SLC6A14 mutants showing nuclei (blue) and gfp (gray). **Bottom;** anti-GFP staining (gray). Scale bars; 20 μm. Red arrows indicate representative localization of SLC6A14. **f**. Growth curves of cells from **(e)**. **g. Left**; [^13^C_3_,^15^N]L-Serine uptake over time of HET293 cells expressing EGFPN1 (EV^EGFPN1^, control), GFP-tagged SLC6A14 WT or V128E, V128G, V128W mutants as indicated. Values are mean±s.e.m. from n = 3 biological replicates. **Right**; Area Under the Curve (AUC) of [^13^C_3_,^15^N]L-Serine uptake curves. Values are mean±s.d. from n = 3 biological replicates. Statistical significance was assessed with ordinary one-way analysis of variance (ANOVA) and Dunnett’s multiple comparisons test. **h. Left;** [^13^C_3_,^15^N]L-Serine uptake after 15 min labeling over increasing concentrations of [^13^C_6_,^15^N]L-Leucine and stable [^13^C_3_,^15^N]L-Serine supply in HCT116 cells overexpressing GFP-tagged SLC6A14. Values are mean±s.d. from n = 3 biological replicates. **Right;** [^13^C_6_,^15^N]L-Leucine uptake after 15 min labeling with increasing concentrations of [^13^C_6_,^15^N]L-Leucine and stable [^13^C_3_,^15^N]L-Serine supply in HCT116 cells overexpressing GFP-tagged SLC6A14. Values are mean±s.d. from n = 3 biological replicates.

To directly decipher the dynamics of SLC6A14-mediated amino acid uptake in HCT116 cells, we exposed NTC (control) or SLC6A14-silenced HCT116 cells to a set of ^13^C/^15^N-labeled amino acids at physiological levels for 1, 3 and 6 min as in previously described uptake assays. Comparing to serine, we used the primary amino acids previously suggested to have facile transport by SLC6A14, namely leucine, isoleucine, valine, methionine, tryptophan and phenylalanine, as well as glutamate and aspartate previously shown not to be transported by SLC6A14 (ref. ^48^). We observed that SLC6A14 silencing in HCT116 cells principally affected uptake of serine with strong effects on methionine, tryptophan, phenylalanine and leucine/isoleucine, but as expected not glutamate or aspartate (Fig. 5b and Extended Data Fig. 4a). We hypothesized that a shortage of intracellular serine induced by *SLC6A14* absence would stimulate serine synthesis and PHGDH, PSAT1 and PSPH seemed approximately 1.5-times higher in cells with constitutive gene deletion of *SLC6A14* (Extended Data Fig. 4b,c). In similarity to results with acute RNAi, constitutive *SLC6A14* knockout decreased the proliferation rate in HCT116 cells (Extended Data Fig. 4d), while overexpression of GFP-tagged SLC6A14 increased proliferation in HCT116 cells (Fig. 5c). Re-expression of SLC6A14–GFP in *SLC6A14*-deleted cells increased consumption of serine compared to empty vector (GFP) controls (Fig. 5d). These results suggest that SLC6A14 is a major serine transporter in these cells.

We next sought to further investigate the relationship between SLC6A14 and serine and whether manipulation of the SLC6A14 molecule influence serine uptake. The structure of SLC6A14 has not yet been experimentally resolved; however, computational tools allow structural modeling with a high confidence score (Fig. 5e). SLC6A14 belongs to the large group of the amino acid-polyamine-organocation (APC) transporters that contain a characteristic LeuT-fold structural domain. The *Aquifex* *aeolicus* Na^+^/amino acid co-transporter LeuT was the first APC member to be structurally resolved at the atomic level^49^. While APC members exhibit major differences in terms of substrate specificity and mechanism, they possess a strikingly similar structure in their LeuT-fold domains. It has been suggested that the amino acid residue in the equivalent position to LeuT V104 controls the space of the binding pocket and when mutated results in major changes in substrate uptake and selectivity in amino acid transporters of the APC superfamily^50^. Our sequence analysis and others^51^ suggest that the V128 of SLC6A14 is the V104 equivalent of LeuT and aligns with LeuT V104-equivalent positions of other distant APC family members that are known to influence substrate selectivity^50^ (Fig. 5f). Sequence alignment suggests that V128 of SLC6A14 is highly conserved within different organisms (Fig. 5g). Recently, V128I (valine to isoleucine) mutation of SLC6A14 had seemed to improve serine uptake in overexpression experiments in *Xenopus* *laevis* oocytes without notable effects on substrate specificity of other amino acids studied. As isoleucine (164 Å^3^) is larger in size than valine (140 Å^3^), perhaps small neutral amino acids such as serine could fit better in this binding pocket; however, the mutation to the even larger phenylalanine (192 Å^3^) did not improve serine uptake and minimized uptake of other amino acids such as arginine and lysine^51^, suggesting that more extensive mutagenesis studies are required to delineate SLC6A14-mediated serine transport.

We investigated further whether the V128 residue of SLC6A14 can control serine uptake by mutating it to the much larger tryptophan (W; 227.8 Å^3^) or to the smaller glycine (G; 60.1 Å^3^) and to the slightly larger but positively charged glutamate (E; 138.4 Å^3^) (Fig. 5h). All mutants still localized to the plasma membrane in HCT116 cells (Extended Data Fig. 4e) but did not alter cell proliferation rates compared to wild-type (WT) SLC6A14 (Extended Data Fig. 4f). Transient overexpression in HEK293 cells of GFP-tagged SLC6A14 (WT) increased [^13^C_3_,^15^N]l-serine uptake compared to empty vector control, whereas mainly V128E and V128W, and to a lesser degree V128G, slowed [^13^C_3_,^15^N]l-serine uptake (Extended Data Fig. 4g). To assess whether SLC6A14 mutants influenced serine dependence and requirements in HCT116 cells, we stably overexpressed them in HCT116 cells and measured [^13^C_3_,^15^N]l-serine consumption rates. While WT SLC6A14 overexpression increased serine consumption from the medium, this behavior was lost in all three mutants studied (Fig. 5i).

As SLC6A14 is reported to have high affinity for leucine^48^, we next assessed potential competition between serine and leucine for SLC6A14-mediated transport and whether increased leucine abundance could influence serine uptake. We supplemented HCT116 stably overexpressing SLC6A14 with increasing, physiologically relevant, concentrations of [^13^C_6_,^15^N]l-leucine (0–400 μM) and a constant concentration of [^13^C_3_,^15^N]l-serine (200 μM) and quantified serine and leucine uptake at a time point where we have observed steady-state serine uptake (15 min) (Extended Data Fig. 4h). Variations in leucine concentration did not alter serine uptake suggesting there is no competition between these two amino acids for SLC6A14 binding at physiological concentrations (Extended Data Fig. 4h). Collectively these results suggest that SLC6A14 has the ability to transport a range of amino acids and functions as a major serine transporter in the studied colorectal cancer cells.

### Dual targeting of serine transporters

**Fig. 6:**
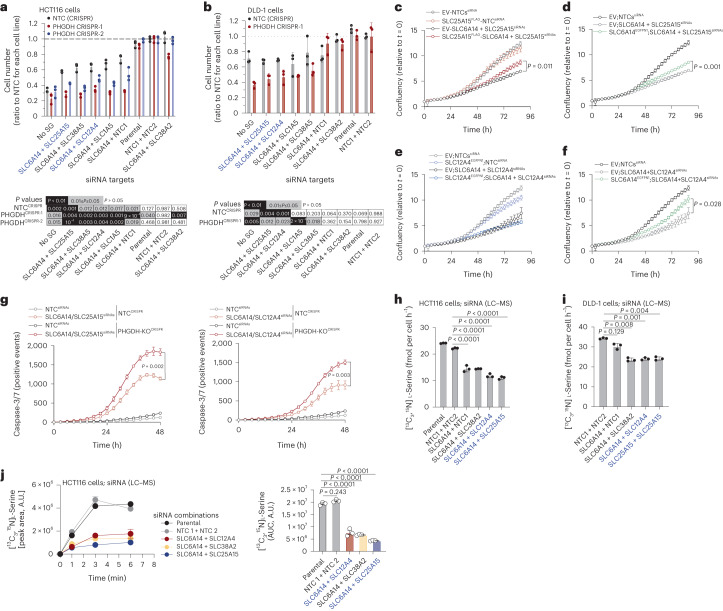
Synthetic lethality between paired serine transporter silencing and PHGDH loss. **a**, Cell number (ratio to NTC) of HCT116 NTC (CRISPR control) and two PHGDH-depleted cell lines upon double knockdown of SLC6A14 and indicated SLC-encoding genes, after 72 h of growth (top). Values are mean ± s.d. from *n* = 3 biological replicates. Statistical significance was assessed by two-tailed one-sample *t*-test on natural log-transformed values. Heat map indicating *P* values for each condition compared to NTC (control) (bottom). **b**, Cell number (ratio to NTC) of DLD-1 NTC (CRISPR control) and PHGDH-depleted cells upon double knockdown of SLC6A14 and indicated SLC-encoding genes, after 72 h of growth (top). Values are mean ± s.d. from *n* = 3 biological replicates. Statistical significance was assessed by two-tailed one-sample *t*-test on natural log-transformed values. Heat map indicating *P* values for each condition compared to NTC (control) (bottom). **c**, Growth curves of HCT116 cells expressing EV (control) or FLAG-tagged SLC25A15 with NTCs (siRNA, control) or silenced for SLC6A14 and SLC25A15. Values are mean ± s.d. from *n* = 3 biological replicates and representative of three independent experiments. Statistical significance assessed by two-tailed Welch’s *t*-test at *t* = 87 h time point. **d**, Growth curves of HCT116 cells expressing EV (control) or GFP-tagged SLC6A14 with NTCs (siRNA, control) or silenced for SLC6A14 and SLC25A15. Values are mean ± s.d. from *n* = 3 biological replicates and representative of three independent experiments. Statistical significance assessed by two-tailed Welch’s *t*-test at *t* = 87 h time point. **e**, Growth curves of HCT116 cells expressing EV (control) or GFP-tagged SLC12A4 with NTCs (siRNA, control) or silenced for SLC6A14 and SLC12A4. Values are mean ± s.d. from *n* = 3 biological replicates and representative of three independent experiments. Control data (EV-NTCs^siRNA^) is replicated in **c**,**d**. **f**, Growth curves of HCT116 cells expressing EV (control) or GFP-tagged SLC6A14 with NTCs (siRNA, control) or silenced for SLC6A14 and SLC12A4. Values are mean ± s.d. from *n* = 3 biological replicates and representative of three independent experiments. Statistical significance assessed by two-tailed Welch’s *t*-test at t = 87 h time point. Control data (EV-NTCs^siRNA^) is replicated in (**c**), (**d**) and (**e**). **g**, Caspase-3/-7-positive events over time of HCT116 NTC (CRISPR control) and PHGDH-depleted cells with NTCs (siRNA, control) or double-silenced for SLC6A14 / SLC25A15 (top) and SLC6A14 / SLC12A4 (bottom). Values are mean ± s.d. from *n* = 3 biological replicates and representative of three independent experiments. Statistical significance assessed by two-tailed Welch’s *t*-test at *t* = 48 h time point. **h**, The [^13^C_3_,^15^N]l-serine consumption in HCT116 cells silenced for SLC6A14 and indicated SLC-encoding genes. Values are mean ± s.d. from *n* = 3 biological replicates. **i**, [^13^C_3_,^15^N]l-Serine consumption in DLD-1 cells silenced for SLC6A14 and indicated SLC-encoding genes. Values are mean ± s.d. from *n* = 3 biological replicates. **j**, The [^13^C_3_,^15^N]l-serine uptake of HCT116 parental, ‘NTC1 and NTC2’ (siRNA control) and cells double-silenced for SLC6A14 and SLC12A4, SLC38A2 or SLC25A15 (left). Values are mean ± s.e.m. from *n* = 3 biological replicates. AUC of [^13^C_3_,^15^N]l-serine uptake curves from left (right). Values are mean ± s.e.m. from *n* = 3 biological replicates. Statistical significance was assessed with ordinary one-way ANOVA and Dunnett’s multiple comparisons test (**h**–**j**).

**Extended Data Fig. 5:**
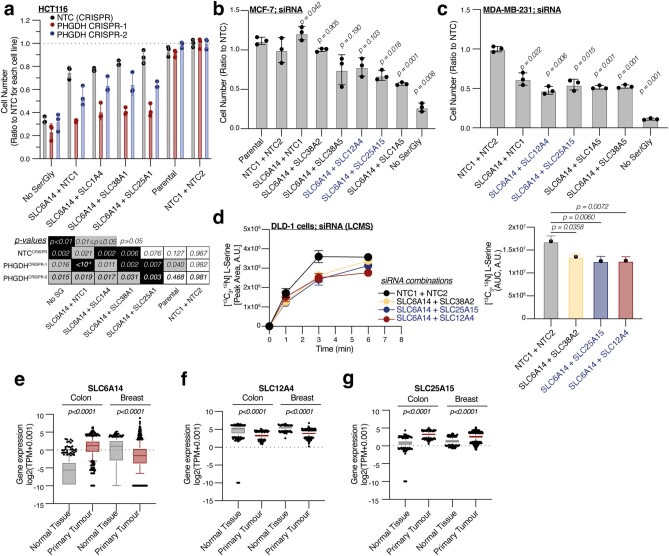
Synthetic lethality between paired serine transporter silencing and PHGDH loss. **a. Top;** Cell number (Ratio to NTC siRNA control) of HCT116 NTC (CRISPR control) and two PHGDH-depleted cell lines upon double knockdown of SLC6A14 and indicated SLC genes, after 72 hours of growth. Values are mean±s.d. from n = 3 biological replicates. Control data (‘NTC1 + NTC2’, ‘Parental’, ‘SLC6A14 + NTC1’ and ‘No Ser/Gly’) are replicated in Fig. 6a. **Bottom;** Heat map indicating *p*-values for each condition compared to NTCs (siRNA control). **b**. Cell number (Ratio to NTC siRNA control) of MCF7 cells upon double knockdown of SLC6A14 and indicated SLC genes, after 72 hours of growth. Values are mean±s.d. from n = 3 biological replicates. **c**. Cell number (Ratio to NTC siRNA control) of MDA-MB-231 cells upon double knockdown of *SLC6A14* and indicated *SLC* genes, after 72 hours of growth. Values are mean±s.d. from n = 3 biological replicates. **d. Left**; [^13^C_3_,^15^N]L-Serine uptake of DLD-1 ‘NTC1 and NTC2’ siRNA (control) and cells double-silenced for SLC6A14 and SLC12A4, SLC38A2 or SLC25A15 as indicated. Values are mean±s.e.m. from n = 3 biological replicates. **Right**; Area Under the Curve (AUC) of [^13^C_3_,^15^N]L-Serine uptake curves from cells on **Left**. Values are mean±s.e.m. and relative to NTC siRNA control from n = 3 biological replicates. Statistical significance was assessed with ordinary one-way analysis of variance (ANOVA) and Dunnett’s multiple comparisons test. **e-g**. Gene expression (log2(TPM + 0.001)) profiles of *SLC6A14* **(e)**, *SLC12A4* **(f)** and *SLC25A15* **(g)** in normal tissue vs primary tumor specimen of colon or breast tissue. Box plots represent median and 25^th^ and 75^th^ percentiles as the limits of the box and whiskers represent 10^th^ and 90^th^ percentiles of the data with individual dots representing outliers from n = 359 normal colon tissue, n = 380 primary colon tumors, n = 292 normal breast tissue and n = 1092 primary breast tumors. Statistical significance was assessed with ordinary one-way analysis of variance (ANOVA) and Dunnett’s multiple comparisons test. No Ser/Gly; no Serine/Glycine condition. Data on a,b,c: Statistical significance was assessed by two-tailed one-sample *t*-test on natural log-transformed values.

As we had identified multiple SLCs capable of serine transport, and that inhibition of individual SLCs caused only a relatively modest decrease in serine uptake, suggesting individual transporter redundancy, we hypothesized that inhibition of two SLCs simultaneously would be more impactful. Dual inhibition of SLC6A14 and either of SLC25A15, SLC38A5, SLC12A4 or SLC1A5, had a greater impact on cell proliferation compared to single SLC6A14 knockdown in HCT116 and DLD-1 cells, and the effect was further enhanced in *PHGDH*-deleted cell lines (Fig. 6a,b). Combination of SLC6A14 with SLC38A2, SLC1A4, SLC38A1 or SLC25A1 failed to elicit greater inhibition than single SLC6A14 knockdown (Fig. 6a and Extended Data Fig. 5a), suggesting that SLC25A15, SLC38A5, SLC12A4 and SLC1A5 are the key transporters cooperating with SLC6A14.

Dual targeting of SLC6A14 with SLC25A15, SLC12A4, SLC1A5 and SLC38A5 also reduced growth in MCF7 and MDA-MB-231 human breast cancer cell lines (Extended Data Fig. 5b,c). Combination of SLC6A14 with SLC1A5 or SLC38A5 had a larger effect in breast versus colorectal cancer cell lines, likely reflecting that tissue of origin can influence SLC substrate selectivity. Notably, we observed higher impact of serine/glycine starvation versus double SLC targeting in these breast cell lines, suggesting that other transporters may contribute to serine uptake in these cells.

Stable overexpression of either FLAG-tagged SLC25A15 or GFP-tagged SLC6A14 in HCT116 cells partially rescued the growth defect conferred by double SLC6A14/SLC25A15 silencing, suggesting that both SLCs are required to fully support proliferation (Fig. 6c,d). Stable overexpression of GFP-tagged SLC12A4 did not rescue growth of SLC6A14/SLC12A4-deficient HCT116 cells, whereas the presence of GFP-tagged SLC6A14 did to a degree (Fig. 6e,f), suggesting, in agreement with our previous data, that SLC12A4 is not a direct carrier of serine but rather possibly facilitates the activity of chloride-dependent transporters such as SLC6A14 through its previously reported involvement in chloride efflux. To assess whether the effect of the combined targeting of serine transporters is cytostatic or induces cell death, we monitored caspase-3/-7 activation in NTC (CRISPR control) or PHGDH-KO HCT116 cells upon SLC6A14/SLC25A15 and SLC6A14/SLC12A4 RNAi-mediated silencing. Independent of PHGDH status, these double combinations induced cell death, a phenotype that was largely enhanced by PHGDH loss (Fig. 6g).

LC–MS confirmed that dual silencing of *SLC6A14* with *SLC12A4*, *SLC25A15* or *SLC38A2* (in the case of DLD-1 cells) decreased serine consumption rates more than single *SLC6A14* knockdown (Fig. 6h,i). In functional serine uptake assays we observed that *SLC6A14/12A4* or *SLC6A14/25A15* or *SLC6A14/38A2* combinations dramatically reduced serine uptake by HCT116 cells (Fig. 6j) and DLD-1 cells (Extended Data Fig. 5d).

Based on these observations we focused on dual inhibition of SLC6A14 with the plasma membrane ion exchanger SLC12A4 or the mitochondrial transporter SLC25A15. To investigate whether these SLCs could have a role in cancer we assessed gene expression levels in healthy tissue or primary tumors of colon or breast origin. *SLC6A14* was upregulated in colon tumors, whereas there was lower expression in breast tumors but with high variance in that group (Extended Data Fig. 5e). While *SLC12A4* showed somewhat decreased expression versus healthy tissue, expression levels were still high in tumors (Extended Data Fig. 5f). *SLC25A15* is upregulated in tumors of colon and breast origin (Extended Data Fig. 5g).

**Extended Data Fig. 6:**
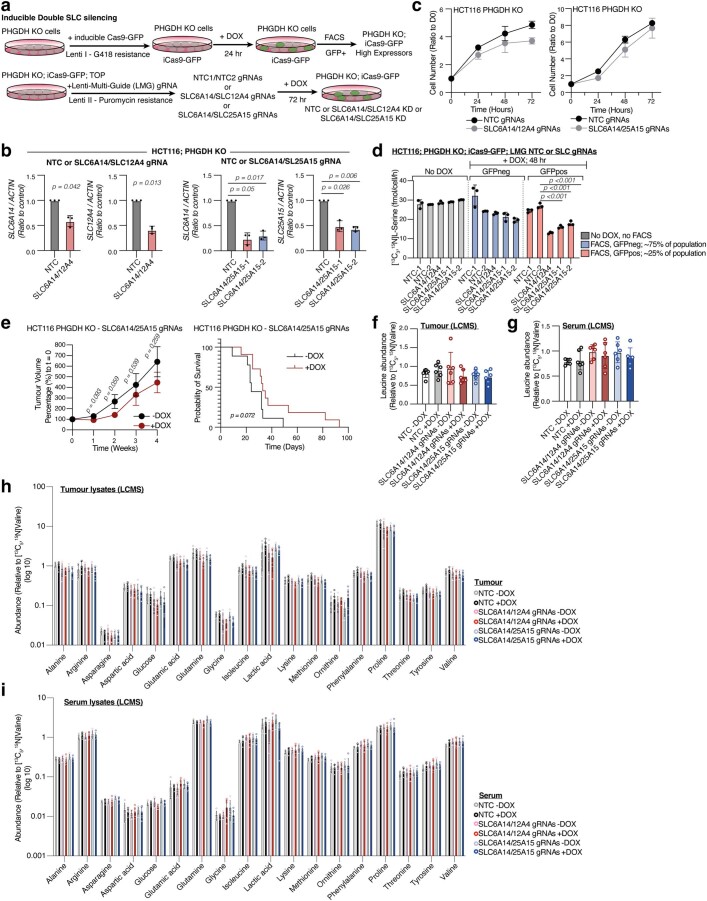
Inducible knockout of *SLC6A14/12A4* and *SLC6A14/25A15* SLC transporters. **a**. Schematic of DOX-inducible iCas9-GFP - NTCs, *SLC6A14/12A4* or *SLC6A14/25A15* gRNAs cell line generation. **b. Left**; mRNA expression levels of *SLC6A14* and *SLC12A4* genes in HCT116 PHGDH-depleted cells expressing iCas9-GFP; NTC or *SLC6A14/SLC12A4* gRNAs following 72 hours of DOX. **Right**; mRNA expression levels of *SLC6A14* and *SLC25A15* genes in HCT116 PHGDH-depleted cells expressing iCas9-GFP; NTC or *SLC6A14/SLC25A15* gRNAs following 72 hours of DOX. Values are mean±s.d. from n = 3 independent experiments. Statistical significance assessed with two-tailed one-sample *t*-test on natural log-transformed values. **c**. Cell number (Ratio to DO) over time (hours) of HCT116 PHGDH-depleted cells expressing iCas9-GFP; NTC or *SLC6A14/SLC12A4* gRNAs **(left)** or NTC or *SLC6A14/SLC25A15* gRNAs **(right)** following 72 hours of DOX. Values are mean±s.d. from n = 3 biological replicates. **d**. [^13^C_3_,^15^N]L-Serine consumption in DOX-inducible iCas9-GFP; PHGDH-depleted HCT116 cells expressing either a combination of NTCs, *SLC6A14/12A4* or *SLC6A14/25A15* gRNAs and treated with ±DOX for 48 hr and FACS sorted for selecting GFPneg and GFPpos populations. Values are mean±s.d. from n = 3 biological replicates. Statistical significance was assessed with ordinary one-way analysis of variance (ANOVA) and Dunnett’s multiple comparisons test comparing the GFPpos ( + DOX 48 hr) group. **e. Left;** Tumor volume over time of tumors from PHGDH-depleted HCT116 cells expressing *SLC6A14/25A15* gRNAs ±DOX. Values are mean±s.e.m. from n = 9 -DOX and n = 11 + DOX mice. Statistical significance was assessed with two-tailed unpaired t-test. **Right;** Kaplan–Meier plot showing probability of survival on mice from **left**. Statistical significance was assessed with Gehan–Breslow-Wilcoxon test. **f**. Leucine levels in tumors formed by PHGDH-depleted HCT116 cells expressing NTC, SLC6A14/SLC12A4 or SLC6A14/SLC25A15 gRNAs ±DOX induction. **g**. Leucine levels in circulating blood serum of mice with tumors formed by PHGDH-depleted HCT116 cells expressing NTC, SLC6A14/SLC12A4 or SLC6A14/SLC25A15 gRNAs ±DOX induction. In **f, g:** Values are mean±s.d. from n = 6 mice per condition. **h**. Levels of detected amino acids, glucose, lactic acid and ornithine in tumors formed by PHGDH-depleted HCT116 cells expressing NTC, SLC6A14/SLC12A4 or SLC6A14/SLC25A15 gRNAs ±DOX induction. **i**. Levels of detected amino acids, glucose, lactic acid and ornithine in circulating blood serum of mice with tumors formed by PHGDH-depleted HCT116 cells expressing NTC, SLC6A14/SLC12A4 or SLC6A14/SLC25A15 gRNAs ±DOX induction. In **h, i:** Values are log-scale of mean±s.d. from n = 6 mice per condition.

To assess the therapeutic relevance of dual targeting SLC6A14/12A4 and SLC6A14/25A15, and considering the lack of specific small molecule inhibitors, we used an inducible CRISPR-Cas9 system to acutely silence two genes simultaneously. We used *PHGDH*-deleted cells, as serine synthesis could compensate for lack of serine uptake and generated inducible Cas9-GFP (iCas9-GFP) cell lines expressing guide RNAs (gRNAs) against *SLC6A14*/*12A4* or *SLC6A14*/*25A15* (Extended Data Fig. 6a). Upon doxycycline (DOX) induction for 72 h we observed decreased expression of *SLC6A14* and *SLC12A4* genes in PHGDH-KO;HCT116^iCas9;SLC6A14/12A4^ and *SLC6A14* and *SLC25A15* genes in PHGDH-KO;HCT116^iCas9;SLC6A14/25A15^ cells (Extended Data Fig. 6b); however, in all cases we detected some degree of maintained expression for these genes, suggesting a substantial fraction of ‘knockout-escaper’ cells, in which iCas9 constructs are not properly activated. Consequently, following 72 h of DOX induction PHGDH-KO;HCT116^iCas9;SLC6A14/12A4^ and PHGDH-KO;HCT116^iCas9;SLC6A14/25A15^ cells showed somewhat lower but similar proliferation to NTC controls (Extended Data Fig. 6c). It is worth noting that NTC gRNAs and +/− DOX groups serve as a control for both off-target effects from Cas9 expression as well as DOX treatment.

**Fig. 7:**
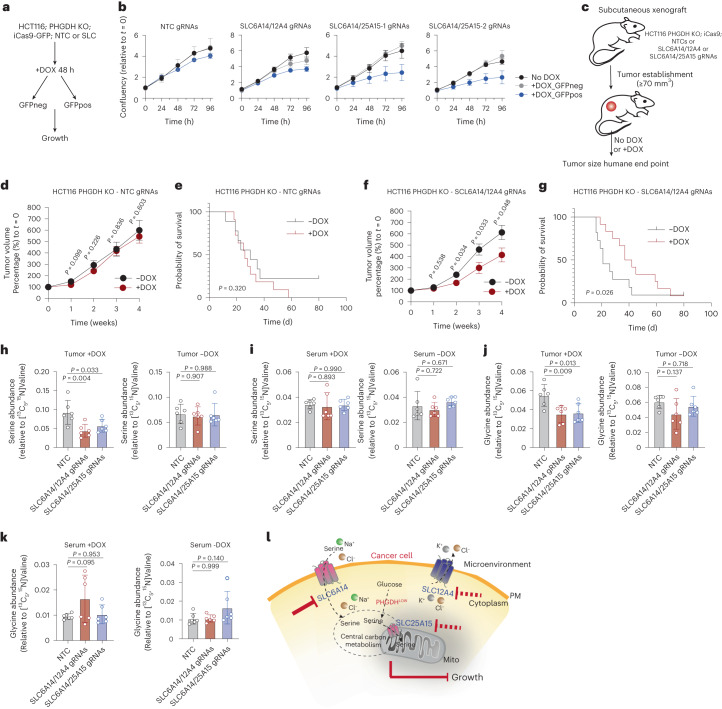
Paired targeting of serine transporters and PHGDH loss impacts colorectal cancer cell growth in vivo. **a**, Schematic of FACS sorting strategy from DOX-inducible iCas9-GFP; PHGDH-depleted HCT116 cells expressing either a combination of NTCs, *SLC6A14/12A4* or *SLC6A14/25A15* gRNAs. **b**, Growth curves of cells from **a**. Data are mean ± s.d. from *n* = 3 biological replicates per condition for each time point. **c**, Schematic of in vivo experiments. **d**, Tumor volume over time of tumors from PHGDH-depleted HCT116 cells expressing NTC gRNAs ±DOX. Values are mean ± s.e.m. from *n* = 9 −DOX and *n* = 11 +DOX mice. **e**, Kaplan–Meier plot showing probability of survival on mice from **d**. **f**, Tumor volume over time of tumors from PHGDH-depleted HCT116 cells expressing *SLC6A14/12A4* gRNAs ±DOX. Values are mean ± s.e.m. from *n* = 11 −DOX and *n* = 12 +DOX treated mice. **g**, Kaplan–Meier plot showing probability of survival on mice from **f**. **h**, Serine levels in tumors formed by PHGDH-depleted HCT116 cells expressing NTC, SLC6A14/SLC12A4 or SLC6A14/SLC25A15 gRNAs with (left) or without (right) DOX induction. Values are mean ± s.d. from *n* = 6 mice per condition. **i**, Serine levels in circulating blood serum of mice with tumors formed by PHGDH-depleted HCT116 cells expressing NTC, SLC6A14/SLC12A4 or SLC6A14/SLC25A15 gRNAs with (left) or without (right) DOX induction. Values are mean ± s.d. from *n* = 6 mice per condition. **j**, Glycine levels in tumors formed by PHGDH-depleted HCT116 cells expressing NTC, SLC6A14/SLC12A4 or SLC6A14/SLC25A15 gRNAs with (left) or without (right) DOX induction. Values are mean ± s.d. from *n* = 6 mice per condition. **k**, Glycine levels in circulating blood serum of mice with tumors formed by PHGDH-depleted HCT116 cells expressing NTC, SLC6A14/SLC12A4 or SLC6A14/SLC25A15 gRNAs with (left) or without (right) DOX induction. Values are mean ± s.d. from *n* = 6 mice per condition. **l**, Schematic summary. Statistical significance was assessed with two-tailed unpaired *t*-test (**d**,**f**), two-tailed Gehan–Breslow–Wilcoxon test (**e**,**g**) and ordinary ANOVA and Dunnett’s multiple comparisons test (**h**–**k**).

To assess whether the lack of impact on proliferation was due to knockout-escapers, we sorted GFP-negative (GFPneg; suggesting escapers) and GFP-positive (GFPpos; suggesting true knockout) populations by FACS after 48 h DOX induction (Fig. 7a). Approximately 25% of cells were GFPpos, indicating that the majority of cells (~75%) are potentially knockout-escapers, losing the capacity to express the iCas9 vector over time. We then monitored the growth and serine consumption of sorted GFPpos and GFPneg cells; GFPpos *SLC6A14*/*12A4* and *SLC6A14*/*25A15* knockouts, but not NTC expressing cells, displayed slower proliferation (Fig. 7b) and lower serine consumption rates (Extended Data Fig. 6d) compared to GFPneg and no-DOX populations.

To investigate the importance of the identified serine transporters in vivo we injected PHGDH-KO;HCT116^iCas9;NTC1/2^ (controls), PHGDH-KO;HCT116^iCas9;SLC6A14/12A4^ and PHGDH-KO;HCT116^iCas9;SLC6A14/25A15^ cells subcutaneously into mice (Fig. 7c). After tumor formation, we treated mice with DOX to induce deletion of *SLC6A14*/*12A4* or *SLC6A14*/*25A15*. Tumors established by NTC cells were not influenced by DOX treatment (Fig. 7d,e). Despite a mixed cell population in terms of DOX responsiveness (Cas9 was predicted to be induced in ~25% of the population, as described above), deletion of *SLC6A14*/*12A4* (Fig. 7f,g), and to a lesser degree *SLC6A14*/*25A15* (Extended Data Fig. 6e), slowed tumor growth and conferred a survival benefit. LC–MS-based metabolomics on tumor lysates demonstrated lower tumor serine and glycine levels upon deletion of *SLC6A14/12A4* and *SLC6A14/25A15* in HCT116 PHGDH-deficient xenografts, whereas serine and glycine levels in the circulating blood remained unaltered (Fig. 7h–k). Also in this in vivo experiment, leucine, a major theoretical substrate of SLC6A14, did not show changes in tumor or serum levels (Extended Data Fig. 6f,g). Furthermore, no major changes were observed in other amino acids (Extended Data Fig. 6h,i). Collectively these results suggest that in serine synthesis-deficient tumors, targeting SLC6A14 together with an ion exchanger/facilitator (SLC12A4) or a mitochondrial serine transporter (SLC25A15) can decrease serine uptake, leading to reduced tumor growth.

**Extended Data Fig. 7:**
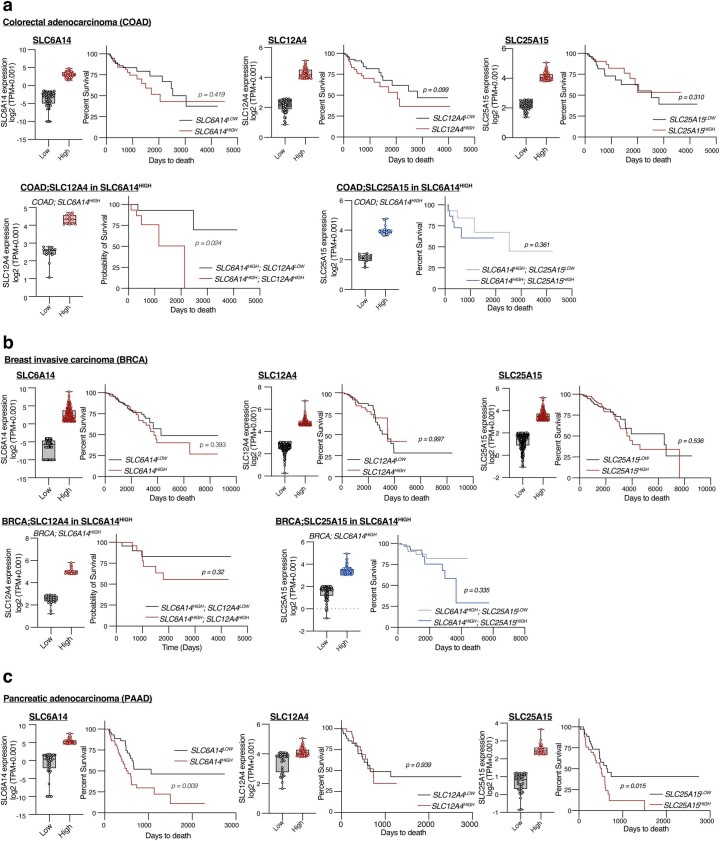
Assessment of the prognostic value of SLC6A14, SLC25A15 and SLC12A4 in cancer patients. **a**. Colon adenocarcinoma (COAD) TCGA dataset: **TOP;** Prognostic value for overall survival of *SLC6A14* transcript levels comparing samples with 25% lowest (*SLC6A14*^*LOW*^*)* (n = 57) and 25% highest (*SLC6A14*^*HIGH*^*)* (n = 80) expression **(left)**, of *SLC12A4* comparing samples with 20% lowest (*SLC12A4*^*LOW*^*)* (n = 58) and 20% highest (*SLC12A4*^*HIGH*^*)* (n = 58) expression **(middle)** and of *SLC25A15* comparing samples with 20% lowest (*SLC25A15*^*LOW*^*)* (n = 58) and 20% highest (*SLC25A15*^*HIGH*^*)* (n = 58) expression **(right)**. **Bottom;** Prognostic value for overall survival of *SLC12A4* transcript levels in *SLC6A14*^*HIGH*^ COAD patients with 20% lowest (SLC6A14^HIGH^;*SLC12A4*^*LOW*^*)* (n = 15) and 20% highest (*SLC6A14*^*HIGH*^*;SLC12A4*^*HIGH*^*)* (n = 15) expression **(left)** and of *SLC25A15* in *SLC6A14*^*HIGH*^ COAD patients with 20% lowest (SLC6A14^HIGH^;*SLC25A15*^*LOW*^*)* (n = 15) and 20% highest (*SLC6A14*^*HIGH*^*;SLC25A15*^*HIGH*^*)* (n = 15) expression **(right)**. **b**. Breast invasive carcinoma (BRCA) TCGA dataset: **TOP;** Prognostic value for overall survival of *SLC6A14* transcript levels comparing samples with 25% lowest (*SLC6A14*^*LOW*^*)* (n = 277) and 25% highest (*SLC6A14*^*HIGH*^*)* (n = 273) expression **(left)**, of *SLC12A4* comparing samples with 15% lowest (*SLC12A4*^*LOW*^*)* (n = 164) and 15% highest (*SLC12A4*^*HIGH*^*)* (n = 164) expression **(middle)** and of *SLC25A15* comparing samples with 25% lowest (*SLC25A15*^*LOW*^*)* (n = 276) and 25% highest (*SLC25A15*^*HIGH*^*)* (n = 273) expression **(right)**. **Bottom;** Prognostic value for overall survival of *SLC12A4* transcript levels in *SLC6A14*^*HIGH*^ BRCA patients with 10% lowest (SLC6A14^HIGH^;*SLC12A4*^*LOW*^*)* (n = 28) and 10% highest (*SLC6A14*^*HIGH*^*;SLC12A4*^*HIGH*^*)* (n = 30) expression **(left)** and of *SLC25A15* in *SLC6A14*^*HIGH*^ BRCA patients with 25% lowest (SLC6A14^HIGH^;*SLC25A15*^*LOW*^*)* (n = 70) and 25% highest (*SLC6A14*^*HIGH*^*;SLC25A15*^*HIGH*^*)* (n = 69) expression **(right)**. **c**. Pancreatic adenocarcinoma (PAAD) TCGA dataset: **TOP;** Prognostic value for overall survival of *SLC6A14* transcript levels comparing samples with 25% lowest (*SLC6A14*^*LOW*^*)* (n = 45) and 25% highest (*SLC6A14*^*HIGH*^*)* (n = 45) expression **(left)**, of *SLC12A4* comparing samples with 20% lowest (*SLC12A4*^*LOW*^*)* (n = 36) and 20% highest (*SLC12A4*^*HIGH*^*)* (n = 36) expression **(middle)** and of *SLC25A15* comparing samples with 20% lowest (*SLC25A15*^*LOW*^*)* (n = 37) and 20% highest (*SLC25A15*^*HIGH*^*)* (n = 40) expression **(right)**. In **a-c**: Box and whiskers plots are log2 (TPM + 0.001) with boxes representing median and 25^th^ and 75^th^ percentiles and whiskers extending from the smallest to the highest value of the data with individual dots showing each point of the dataset. Kaplan–Meier curves were statistically compared using log-rank (Mantel^−^Cox) test.

Analysis of colon adenocarcinoma (COAD) primary tumor TCGA datasets revealed that high *SLC6A14*, *SLC12A4* or *SLC25A15* expression when evaluated individually are not predictive of overall survival; however, when coexpression of two SLCs of interest were evaluated, patients with high *SLC6A14* and high *SLC12A4* had significantly shorter survival than those with high *SLC6A14* and low *SLC12A4* (Extended Data Fig. 7a), suggesting a potential functional link and predictive signature for these two transporters. No predictive value was observed by *SLC6A14*-high and *SLC25A15*-high expression in the COAD dataset (Extended Data Fig. 7a). In breast invasive carcinoma no predictive value was observed by *SLC6A14*, *SLC12A4* or *SLC25A15* alone, but there was a nonsignificant trend for reduced survival in *SLC6A14*-high combined with *SLC12A4*-high or *SLC25A15*-high expression (Extended Data Fig. 7b). In patients with pancreatic adenocarcinoma (PAAD), *SLC6A14* or *SLC25A15* expression alone seemed highly prognostic for lower overall survival (Extended Data Fig. 7c), potentially reflecting an advantage of extracellular serine consumption in the nutrient-deprived microenvironment of this tumor type^4^.

In summary (Fig. 7l), our experiments identified a plasma membrane serine transporter (SLC6A14), a serine transport facilitator (SLC12A4) and a mitochondrial serine transporter (SLC25A15). Our data suggest that targeting serine uptake by blocking a main serine plasma membrane amino acid transporter together with a facilitator of amino acid transport (in this case an ion exchanger) or a mitochondrial serine transporter, confers therapeutic benefit in serine synthesis-deficient cancer cells.

## Discussion

Amino acid transport in mammalian cells is primarily mediated by secondary active transporters of the SLC superfamily. Principally these are symporters, where amino acid transport is coupled to co-transport of ions such as Na^+^, Cl^−^, H^+^ or K^+^, or they are antiporters where one amino acid is exchanged for another^21^. While a remarkable amount of information exists on basic SLC biochemistry, potential substrate affinity and mechanism of transport, most arises from overexpression of cloned transporter sequences in systems such as *Xenopus* oocytes or bacteria, which are then analyzed by electrophysiology or radiolabeling assays in nonphysiological buffers; however, to understand their true biological functions, studies within an endogenous cellular context should be performed. In addition to technical limitations, exploration of SLC roles is further complicated by overlapping substrate specificities between SLC members, but also by the impact of environmental and tissue-specific cues on their substrate affinity, localization, expression and regulation. It is likely that differences not only in the absolute concentration of substrates but also in their relative abundance in the microenvironment would influence SLC affinity, whereas different cell types may be equipped by different SLC palettes for the same substrates. The process of tumor growth is likely to have distinct influences on SLC expression and function, as physiological amino acid homeostasis mechanisms are perturbed.

Recent studies have emerged aiming to discern amino acid transport by SLCs in more physiological systems. By using a combination of radiolabeled amino acid uptake assays and computational modeling of certain known amino acid transporters, it has been suggested that SLC amino acid transporters are the principal determinants of intracellular amino acid levels and that combined action of different SLCs (such as uniporters with antiporters) may maintain homeostasis of amino acid pools in physiology^52^. Recently, a CRISPR/Cas9-activation screening of SLC genes in HEK293 cells revealed transporter–nutrient relationships in nutrient-limiting conditions. Individual depletion of 13 amino acids revealed a range of SLC responses upon nutrient limitation and showed that SLC upregulation is an adaptation to nutrient-restrictive conditions. Notably, SLC6A14 seemed to control cell survival upon restriction of each of the studied amino acids. It is also notable that this screen was performed in cells with an intact serine synthesis pathway, potentially explaining why serine/glycine restriction did not cause large enrichment of SLC genes^33^.

In the present study, we designed an assay medium containing all proteinogenic amino acids at the upper end of physiological serum concentration ranges; thus, cancer cells, which rapidly consume available nutrients in closed culture systems, may not fully deplete nutrients from the medium during experiments. Furthermore, cells were exposed to potential substrates at physiological relative ratios. We also selected an acute RNAi approach to assess SLC–nutrient relationships, as cells may compensate for constitutive SLC loss induced by chronic CRISPR/Cas9 deletion. Results from our arrayed RNAi/LC–MS screen revealed notable changes in exogenous amino acid levels following silencing of SLC genes. Specifically, most SLCs seemed to impact glutamine uptake. With numerous roles in anabolic metabolism and stress response, cancer cells generally have higher demands for glutamine than any other amino acid^28^. We observed that when glutamine uptake was decreased, the uptake of other amino acids increased. This potentially stems from the induced need to synthesize glutamine intracellularly which requires nitrogen available from other exogenous amino acids. Likewise, inhibition of tyrosine uptake could stimulate the uptake of its precursor phenylalanine (essential amino acid) and inhibition of cysteine/cystine uptake could stimulate methionine (essential amino acid) uptake for the same reason.

Another logical explanation for observing increased amino acid uptake in this assay is that antiporter function may be disrupted by changing import of a given amino acid. For example, the xCT system (SLC7A11) imports cystine while exporting glutamic acid^53^. Inhibition of this system is predicted to increase extracellular cysteine levels but to decrease extracellular glutamic acid levels. Also, upon knockdown, many SLCs induced increased general uptake of amino acids, whereas others decreased uptake of some amino acids and increased others. While these results may suggest antiporter/efflux roles for some SLCs, these data should be interpreted carefully; it reflects both the direct effects on amino acid transport caused by SLC knockdown, but also how the resulting effects on cell proliferation or other cellular needs may subsequently alter global nutrient demands. To account for this potential confounding factor in studying serine transport, we employed matched serine-auxotrophic and non-auxotrophic cell lines to select SLC members that primarily influence serine uptake. By profiling amino acid consumption and growth patterns after RNAi-mediated silencing of the SLC superfamily, we shortlisted the SLC members involved in serine transport in cancer cells.

Using physiologically relevant conditions, we found that SLC6A14 is a plasma membrane serine transporter and SLC25A15 a mitochondrial serine transporter in cancer cells. While in less physiological settings these SLCs have been shown to favor other substrates, our data suggest that they drive serine uptake in the cytosol and mitochondria of colorectal cancer cells. SLC12A4 strongly influenced serine uptake, potentially by a role in ion exchange. Considering that SLC6A14 is thought to be a Na^+^/Cl^−^ dependent transporter, and SLC12A4 is known to export K^+^/Cl^−^, it is logical to speculate that SLC12A4 supports SLC6A14 activity by buffering Cl^−^ levels. Future work is required to precisely elucidate the exact dynamics of this relationship and how alterations in ion homeostasis influence nutrient uptake/release in pathophysiology.

We also identified other SLC amino acid transporters, such as SLC38A2 and SLC1A5, with the potential to mediate serine transport but they did not seem essential for this process in colorectal cancer cells. It is possible, however, that in other cell lines and tumor types they may play a more dominant role in serine transport. Furthermore, while SLC6A14 seemed to be a primary serine transporter in our study, it also clearly transported most neutral amino acids. Further studies are required to establish whether serine is a primary SLC6A14 substrate in different contexts or whether it switches to other amino acids in alternative conditions. Identifying the palette of transporters that are employed by different tissues or between cell types within the same tissue is a challenge for the immediate future and could lead to improved understanding of nutrient networks in tissue and tumor microenvironments. Our results show that dual targeting of SLC6A14 with SLC25A15 or SLC12A4 is detrimental to cancer cell proliferation, especially in the context of low de novo serine synthesis.

A limitation of the present study is that it is restricted to SLC-mediated serine uptake; we did not evaluate other sources of extracellular serine, such as extracellular vesicle uptake or protein scavenging for example by macropinocytosis. Nevertheless, in established serine synthesis-deficient tumor xenografts, acute silencing of SLC6A14/SLC12A4 and to a lesser degree SLC6A14/SLC25A15 reduced tumor growth and serine levels, despite a large proportion of DOX escapers. Development of specific small molecule inhibitors for these SLCs would allow more complete interrogation of their therapeutic potential. SLCs are emerging as promising targets for the treatment of cancer (SLC7A4 (refs. ^54,55^), SLC1A5 (refs. ^56,57^) and SLC7A5 (ref. ^58^)) and other diseases such as diabetes (SLC5A1/SLC5A2 (ref. ^59^)). Further research is required to elucidate the specific regulatory mechanisms of the shortlisted SLCs and their broader roles across tumor types and healthy tissue.

## Methods

### Cell culture

All cell lines used in this study were cultured at 37 °C in 5% CO_2_ in a humidified incubator. Human cell lines were authenticated by STR profiling using Promega GenePrint 10 and tested for *Mycoplasma* using Mycoalert (Lonza). Other than HCT116 p21^−^^/^^−^ (a gift of B. Vogelstein^60^) all cell lines were obtained from the ATCC. HCT116 (CCL-247), HCT116 p21^−^^/^^−^, MCF7 (HTB-22), MDA-MB-231 (HTB-26), HEK293 (CRL-1573) *Homo* *sapiens* cells and COS7 (CRL-1651) *Cercopithecus* *aethiops* kidney cells were cultured in DMEM (Thermo Fisher Scientific, Gibco, 21969035) supplemented with 10% FBS (Thermo Fisher Scientific, Gibco, 10500064), penicillin–streptomycin (10,000 U ml^−^^1^, Thermo Fisher Scientific, Gibco, 15140122), amphotericin B (0.5 μg ml^−^^1^, Thermo Fisher Scientific, Gibco, 15290026) and l-glutamine (2 mM, Thermo Fisher Scientific, Gibco, 25030024). DLD-1 (CCL-221) *H.* *sapiens* cells were cultured in RPMI 1640 (Thermo Fisher Scientific, Gibco, 31870025) supplemented with 10% FBS (Thermo Fisher Scientific, Gibco, 10500064), penicillin–streptomycin (10,000 U ml^−^^1^, Thermo Fisher Scientific, Gibco, 15140122), amphotericin B (0.5 μg ml^−^^1^, Thermo Fisher Scientific, Gibco, 15290026) and l-glutamine (2 mM, Thermo Fisher Scientific, Gibco, 25030024).

For serine and glycine starvation assays, MEM (Thermo Fisher Scientific, Gibco, 21090022) was used as base medium supplemented with l-glutamine (2 mM, Thermo Fisher Scientific, Gibco, 25030024), MEM Vitamin Solution (1%, Merck, Sigma-Aldrich, M6895), 10% dialyzed FBS (Thermo Fisher Scientific, Gibco, 26400044), penicillin–streptomycin (10,000 U ml^−^^1^, Thermo Fisher Scientific, Gibco, 15140122), amphotericin B (0.5 μg ml^−^^1^, Thermo Fisher Scientific, Gibco, 15290026) and adjusted with glucose solution (Thermo Fisher Scientific, Gibco, A2494001) to 15.5 mM glucose. This base medium was also supplemented with serine (0.4 mM) and/or glycine (0.4 mM) and/or formic acid (Merck, Sigma-Aldrich, F0507).

For experimental assays, we formulated a medium (referred as ‘assay medium’) containing all amino acids at physiological concentrations (based on the upper range of serum blood concentration as shown in the Human Metabolome Database^61^), specifically: l-histidine (0.1 mM), l-isoleucine (0.1 mM), l-leucine (0.15 mM), l-lysine (0.22 mM), l-phenylalanine (0.1 mM), l-threonine (0.15 mM), l-tryptophan (0.05 mM), l-valine (0.23 mM), l-arginine (0.1 mM), l-glutamine (0.6 mM), l-tyrosine (0.1 mM), l-alanine (0.35 mM), l-proline (0.2 mM), l-glutamate (0.1 mM), l-aspartate (0.04 mM), l-asparagine (0.05 mM), l-cysteine (0.1 mM), l-serine (0.2 mM), glycine (0.2 mM), l-methionine (0.05 mM), cystine (0.1 mM), hydroxyproline (0.02 mM), pyruvate (0.065 mM), l-lactate (1 mM) and d-glucose (12 mM). In Fig. 4, the assay medium was supplemented with l-ornithine (0.2 mM). This experimental medium was supplemented with 10% dialyzed FBS (Thermo Fisher Scientific, Gibco, 26400044), MEM Vitamin Solution (1%, Merck, Sigma-Aldrich, M6895), penicillin–streptomycin (10,000 U ml^−^^1^, Thermo Fisher Scientific, Gibco, 15140122) and amphotericin B (0.5 μg ml^−^^1^, Thermo Fisher Scientific, Gibco, 15290026).

### RNAi-mediated gene silencing

For the initial screen, a library of pooled four siRNA oligonucleotides per target was used against SLC-encoding genes (Horizon Discovery, ON-TARGETplus). For subsequent validation and functional characterization experiments, a single deconvoluted siRNA was used per target (Horizon Discovery, ON-TARGETplus) with the highest silencing efficiency as evidenced by gene expression analysis (Sequence information on Supplementary Table 2). siRNA-mediated silencing was performed with 25 nM siRNA (Horizon Discovery) of either SLC-encoding genes or NTC siRNA (Horizon Discovery). siRNA oligonucleotides were introduced to cells with the Lipofectamine RNAiMax transfection reagent (Thermo Fisher Scientific, Invitrogen, 13778075) according to the manufacturer’s instructions. Cells were typically analyzed 72 h post-transfection.

### Cloning

Codon-optimized open reading frame sequences of *H.* *sapiens SLC6A14*, *SLC12A4*, *SLC25A15*, *SLC38A2*, *SLC38A5* and *SLC1A5* were from the following plasmids; pDONR221_SLC6A14 (Addgene, 131865), pDONR221_SLC12A4 (Addgene, 131932), pDONR221_SLC25A15 (Addgene, 131967), pDONR221_SLC38A2 (Addgene, 132059), pDONR221_SLC38A5 (Addgene, 132118) and pDONR221_SLC1A5 (Addgene, 131974), which were a gift from the RESOLUTE Consortium and G. Superti-Furga. Open reading frame sequences were PCR amplified and cloned into pEGFP-C1, pEGFP-N1 and pcDNA3.1(+)-FLAG vectors using the Gibson assembly kit (NEB) according to the manufacturer’s instructions. DNA construct information is provided in Supplementary Table 3.

### Site-directed mutagenesis

Mutagenesis primers were designed using the NEBaseChanger website (v.1.2.6) and point mutations were generated within the cDNA of *SLC6A14* (*H.* *sapiens*) using the Q5-site-directed mutagenesis kit according to the manufacturer’s instructions (NEB, EO554). The following primers were used to introduce the indicated mutations in the EGFPN1–SLC6A14 (*H.* *sapiens*) construct: V128G, Fw-5′-CTC CAT CTT TGG GAC CAT CTA CT-3′, Rv-5′-ATC AGC ACC ATT GTG ATG-3′. V128E, Fw-5′-CTC CAT CTT TGA GAC CAT CTA CTA TAA C-3′, Rv-5′-ATC AGC ACC ATT GTG ATG-3′. V128W, Fw-5′-CTC CAT CTT TTG GAC CAT CTA CTA TAA C-3′, Rv-5′-ATC AGC ACC ATT GTG ATG -3′.

### Transfection of DNA constructs

Typically, 3 × 10^5^ cells per well were plated on six-well plates. After 16 h, 0.5–1 μg of plasmid DNA was mixed with 5 μl Lipofectamine 2000 transfection reagent (Thermo Fisher Scientific, 11668019) in serum-free medium to a total volume of 200 μl. Following incubation at room temperature for 5 min, DNA/Lipofectamine 2000 mix was added to cells. Transfection efficiency and phenotypic analyses were performed 48 h post-transfection.

Regarding SLC6A14–EGFPN1 (WT, V128E, V128G and V128W), SLC12A4-EGFPN1 and SLC25A15–FLAG constructs, HCT116 cells were transfected with 5 μg DNA using Amaxa Cell Line Nucleofector kit (Lonza Bioscience) according to the manufacturer’s instructions. Stable HCT116 expressors of the above constructs were selected by using puromycin (4 μg ml^–1^) followed by FACS, gating for cell size, live/dead staining and GFP-positive signal (for SLC6A14-EGFPN1 (WT, V128E, V128G and V128W) and SLC12A4–EGFPN1-expressing HCT116 cells).

### CRISPR-mediated gene knockout

Single-guide RNA oligonucleotides were designed using the DKFZ E-CRISPR design tool (www.e-crisp.org/E-CRISP/designcrispr.html). The following sgRNAs were used either targeting *PHGDH*, 5′-TGC AAG ATC TTC CGG CAG CA-3′ and 5′-TGC CGG AAG ATC TTG CAA GA-3′, *SLC6A14* or NTC, 5′-AAA ATA GCA GTA AAC TCA AC-3′. Annealed oligonucleotides were cloned into pSpCas9(BB)-2A-Puro (PX459) V2.0 (a gift from F. Zhang, Addgene, 62988). Cells were transfected with 5 μg of selected plasmid (control, NTC or containing gRNA against *PHGDH*) using Amaxa Cell Line Nucleofector kit (Lonza Bioscience) according to the manufacturer’s instructions. Following selection using puromycin (4 μg ml^–1^), clonal colonies were selected on the basis of efficient PHGDH knockout. DNA construct information is provided in Supplementary Table 3.

### Inducible CRISPR-mediated gene silencing

HCT116 *PHGDH*-KO cells were transduced using a lentivirus-mediated approach to express a DOX-inducible Cas9 plasmid (a gift from Q. Yan, Lenti-iCas9-neo, Addgene, 85400)^62^. Specifically, lentivirus production was in HEK293 cells plated in 10-cm dishes by co-transfection of Lenti-iCas9-neo plasmid with the packaging plasmids psPAX2 and pVSV-G. At 24 h post-transfection, the medium was refreshed and at 48 h post-transfection, virus-containing supernatants were collected, passed through a 0.45-μm filter and mixed with 10 μg ml^−1^ Polybrene. Recipient cells were exposed to viral supernatants for two 24-h periods before selection with geneticin (0.6 mg ml^−^^1^). Selected cells were exposed to 1 μg ml^−^^1^ DOX for 24 h and EGFP-positive cells were sorted by FACS, selecting cells with high Cas9 induction efficiency (referred to as HCT116 PHGDH-KO; iCas9).

sgRNAs against *SLC6A14*, *SLC12A4*, *SLC25A15* or NTC were designed using the DKFZ E-CRISPR design tool (www.e-crisp.org/E-CRISP/designcrispr.html). gRNA sequences used were NTC1, 5′-AAA ATA GCA GTA AAC TCA AC-3′; NTC2, 5′-GAA GAA GAA TTG GGG ATG ATG-3′; *SLC6A14*, 5′-TCA GTA AAG TGG CGC TCC AA-3′; *SLC12A4*, 5′-AGA GCT GGA CAT CCG CCC AA-3′; *SLC25A15*, gRNA_1 5′- GGC TTC CGT GGC TTC TAC AA-3′, gRNA_2 5′- GGC ACT TCA CGA GCT CCG TG-3′. The lenti-multi-CRISPR plasmid (a gift from Q. Yan, Addgene, 85402) was used to express two single gRNA cassettes^62^, specifically NTC1/NTC2, *SLC6A14*/*SLC12A4* and *SLC6A14/SLC25A15* combinations. These were introduced to HCT116 PHGDH-KO; iCas9 through a second round of lentiviral transduction followed by antibiotic selection with 4 μg ml^−1^ puromycin. Gene silencing was induced with 2 μg ml^−^^1^ DOX for 72 h. DNA construct information is provided in Supplementary Table 3.

### Alignment and homology modeling

Predicted structures for human SLC6A14 were taken from the AlphaFold protein structure database (alphafold.ebi.ac.uk/)^63,64^. A full-length protein sequence alignment of LeuT-containing transporters was performed using PROMALS3D multiple sequence and structure alignment server^65^. Alignment of multiple sequences corresponding to SLC6A14 across different organisms was performed using Clustal Omega tool (EMBL-EBI)^66,67^.

### Cell number measurements and growth curves

Typically, 7,000 cells per well were plated on 96-well plates and transfected with siRNA as described above. After 72 h of gene silencing, cells were washed with PBS and fixed for 10 min with 4% formaldehyde. Cells were then washed with PBS and permeabilized with 0.01% Triton in PBS. Cells were stained with 4,6-diamidino-2-phenylindole (DAPI; 1 μg ml^−^^1^ Thermo Fisher Scientific, 62248) for 10 min followed by washes in PBS. Plates were imaged using an Operetta high-content imaging system (PerkinElmer) equipped with a ×10/0.30 LWD (HH12000502) objective and Harmony (PerkinElmer, v.4.1) and cell numbers per well were quantified using an automated analysis pipeline in the Columbus Image Data Storage and Analysis System (PerkinElmer, v.2.8.0).

To generate growth curves, 20,000 cells were typically plated on 24-well plates and allowed to adhere overnight. Adherent cells were washed twice with PBS and were fed with assay medium. A separate counting plate was used to record cell number at *t* = 0. The medium was refreshed every 24 h and plates were fixed at respective timepoints as stated on each figure. Plates were stained with DAPI as described above. All plates were imaged using an Operetta high-content imaging system (PerkinElmer) equipped with a ×10/NA 0.30 (HH12000502) LWD objective and Harmony (PerkinElmer) and cell numbers per well were quantified using an automated analysis pipeline in the Columbus Image Data Storage and Analysis System (PerkinElmer). Relative cell number was calculated as a ratio to *t* = 0.

In Figs. 5c and 6c–f and Extended Data Fig. 4f, typically 4,000 cells were plated per well on 96-well plates (Greiner Bio-One, 655090) and after 6 h, plates were transferred into an Incucyte SX5 Live-cell imaging system (Sartorius). In Fig. 6c–f, cells were also transfected with siRNA as indicated on the figures, using Lipofectamine RNAiMax as described above. Images were typically acquired at 180-min intervals for a period of 96 h and confluence per well was quantified in an automated manner using the Incucyte Live-cell imaging and analysis software (v.2022A, Sartorius). In Fig. 7b, cells were plated as described above and were imaged and analyzed using Incucyte S3 Live-cell imaging system and analysis software (v.2021A, Sartorius).

### Caspase-3/-7 activation assay

A total of 6,000 cells were plated per well on 96-well plates (Greiner Bio-One, 655090) and transfected with siRNA as indicated, using Lipofectamine RNAiMax as described above. The medium was supplemented with the NucView-488 Caspase-3/-7 activation reporter (5 μM) (synthesized by the Chemical Biology STP, The Francis Crick Institute)^68^. After 6 h plates were transferred into an Incucyte SX5 Live-cell imaging system (Sartorius). Images were typically acquired at 180-min intervals for a period of 96 h and GFP-positive events were quantified in an automated manner using Incucyte Live-cell imaging and analysis software (v.2022A, Sartorius).

### Quantification of gene expression

RNA was isolated from 24- or 6-well plates using the RNeasy Plus Mini kit (QIAGEN, 74136) combined with RNase-Free DNase (QIAGEN, 79254) treatment, according to the manufacturer’s instructions. Measurements of RNA concentration and purity were routinely performed using NanoDrop2000C (Thermo Fisher Scientific) before downstream processing. cDNA synthesis was performed with the QuantiTect Reverse Transcription kit (QIAGEN, 205311) using typically 0.5 μg of RNA. Quantitative PCR with reverse transcription (qRT–PCR) analysis was performed in technical triplicate using QuantStudio 7 Flex system (Applied Biosystems, Thermo Fisher Scientific) with the Fast SYBR Green Master Mix (Thermo Fisher Scientific, Invitrogen, 4385612) and data were acquired with the QuantStudio Real-Time PCR software v.1.7.2 (Applied Biosystems, Thermo Fisher Scientific). Relative mRNA quantification was performed using the 2^Δ^^Δ^^CT^ method for multiple genes. A list of the qRT–PCR primer sequences is provided in Supplementary Table 4.

### Mitochondrial/cytosol fractionation for protein extraction

Cells were cultured in 15-cm dishes (>90% confluence). Plates were washed twice in ice-cold PBS and cells were collected in 0.5 ml ice-cold PBS. Following centrifugation at 1,000*g* for 1 min at 4 °C, the supernatant was removed and cells were incubated at 4 °C for 20 min in a hypotonic mitochondrial fractionation buffer (20 mM HEPES, 3 mM EDTA, 250 mM Sucrose) supplemented with protease (cOmplete, EDTA-free Protease Inhibitor Cocktail, Roche, Merck, 4693159001) and phosphatase (PhosSTOP, Roche, Merck, 4906845001) inhibitors. Cells were carefully homogenized using a Dounce homogenizer (20 passes). Lysates were then clarified by sequential centrifugations; after centrifugation for 10 min at 1,200*g* at 4 °C, the supernatant was collected and further centrifuged at 16,600*g* for 10 min at 4 °C. The supernatant was retained as the cytosolic fraction. The pellet containing the mitochondrial fraction was resuspended in mitochondrial lysis buffer containing 1% Triton and further centrifuged at 17,000*g* for 10 min. The supernatant was kept containing solubilized mitochondrial proteins.

### Western blotting

Proteins were extracted in RIPA Lysis and Extraction buffer (Thermo Fisher Scientific, 89900) supplemented with protease (cOmplete, EDTA-free Protease Inhibitor Cocktail, Roche, Merck, 4693159001) and phosphatase (PhosSTOP, Roche, Merck, 4906845001) inhibitors. Lysates were cleared by centrifugation at 18,000*g* for 10 min at 4 °C. Total protein content was quantified by Pierce BCA assay (Thermo Fisher Scientific, 23227). Following normalization to total protein content and addition of 4× Bolt LDS Sample Buffer (+355 mM β-mercaptoethanol), lysates were heated to 70 °C for 10 min and typically 20 μg proteins were resolved on Bolt 4–12% Bis-Tris polyacrylamide gels (Thermo Fisher Scientific, NW04122BOX) using Bolt MOPS SDS Running Buffer (Thermo Fisher Scientific, B0001) and transferred to nitrocellulose membranes (Thermo Fisher Scientific, 88018). Membranes were blocked for 60 min using Intercept (TBS) Blocking Buffer (LI-COR, 927-60001) and primary antibodies against PHGDH (1:1,000 dilution; Cell Signaling Technologies, 13428), PSAT1 (1:1,000 dilution; Novus Biologicals, NBP1-32920), PSPH (1:1,000 dilution; Santa Cruz Biotechnology, sc-98683), SLC25A15 (1:1,000 dilution, Abcam, ab228604), UQCRFS1 (1:1,000 dilution, ProteinTech 18443-1-AP), GAPDH (1:2,000 dilution, Merck/Millipore MAB374) and ACTIN (1:10,000 dilution; Merck/Millipore, MAB1501) were incubated overnight at 4 °C in blocking solution. Membranes were washed three times in TBS + 0.025% Tween-20 (Merck, Sigma-Aldrich, P7949) and incubated with secondary antibodies IRDye 680RD donkey anti-rabbit IgG (1:10,000 dilution; LI-COR, 92568073) and IRDye 800CW donkey anti-mouse IgG (1:10,000 dilution; LI-COR, 92532212) for 60 min at room temperature and washed three times in TBS + 0.025% Tween-20. Fluorescence signal was captured and quantified using a LI-COR Odyssey Fc Imaging System (LI-COR Biosciences) with Image Studio software (v.5.2).

For Fig. 4e, Extended Data Fig. 1d–f and Extended Data Fig. 3i, following normalization to total protein content (as described above), NuPAGE LDS Sample Buffer (4×) (Thermo Fisher Scientific, NP0007) supplemented with Bond-Breaker TCEP Solution, Neutral pH (10×) (Thermo Fisher Scientific, 77720) was added to the lysates, which were then incubated at 37 °C for 20 min. Typically 40 μg proteins were resolved on NuPAGE 4–12% Bis-Tris polyacrylamide gels (Thermo Fisher Scientific, NP0336BOX) or NuPAGE 3–8%, Tris-acetate protein gels (Thermo Fisher Scientific, EA03755BOX) using NuPAGE MOPS SDS Running Buffer (Thermo Fisher Scientific, NP000102) or NuPAGE Tris-acetate SDS Running Buffer (Thermo Fisher Scientific, LA0041), respectively and transferred to nitrocellulose membranes (Amersham Protran 0.45 NC nitrocellulose western blotting membranes, Cytiva, 10600007). Membranes were blocked for 60 min using 5% milk in TBS + 0.025% Tween-20 and probed overnight at 4 °C with primary antibodies against SLC12A4 (1:1,000 dilution; Thermo Fisher Scientific, PA5-77471), SLC6A14 (1:1,000 dilution; Thermo Fisher Scientific, PA5104151), SLC25A15 (1:1,000 dilution; Abcam, ab228604) and vinculin (1:2,000 dilution; Santa Cruz, sc-73614). Membranes were washed three times in TBS + 0.025% Tween-20 and incubated with anti-mouse IgG or anti-rabbit IgG HRP-linked secondary antibodies (1:1,000 dilution; Cell Signaling Technology 7074, 7076) for 60 min at room temperature and washed three times in TBS + 0.025% Tween-20. Blots were developed with Pierce ECL chemiluminescence kit (Thermo Fisher Scientific, 32106) using a tabletop developer (Colenta, MP900e).

Antibody information is provided in Supplementary Table 5.

### Immunofluorescence

Following 24 h of transfection with cDNA, 10,000 cells were plated on 96-well plates coated with fibronectin (10 μg ml^−^^1^) and left to adhere overnight. Cells were washed with PBS and fixed with 4% formaldehyde for 10 min. They were then permeabilized with 0.1% Triton X-100 for 5 min and incubated for 60 min with the following primary antibodies: anti-FLAG (DYKDDDDK) (1:500 dilution; Cell Signaling Technology, 2947), anti-GFP (1:2,000 dilution; Abcam, ab13970) and anti-SLC6A14 (1:500 dilution; St John’s Laboratory, STJ112596). Detection was performed using the following secondary antibodies: Alexa Fluor 488 donkey anti-rabbit (1:500 dilution; Invitrogen, A21206), Alexa Fluor 594 donkey anti-mouse (1:500 dilution; Invitrogen, A21203) or Alexa Fluor 488 goat anti-chicken (1:500 dilution; Invitrogen, A11039). Nuclei were visualized with DAPI (1 μg ml^−^^1^ Thermo Fisher Scientific, 62248) and F-actin with Alexa Fluor 647 Phalloidin (1:100 dilution; Invitrogen, A22287). Images were acquired using the Opera Phenix High-Content Screening System (PerkinElmer) equipped with a ×63/1.15 water (HH14000423) objective and Harmony (PerkinElmer, v.4.9). Images in Extended Data Fig. 4e were acquired using a Zeiss AxioImager.M1 equipped with a Zeiss Plan-Apochromat ×100 1.4 oil DIC objective and using MicroManager (v.2.0)^69^ software for acquisition. All images were processed with Fiji software (ImageJ v.2.0.0). Antibody information is provided in Supplementary Table 5.

### [^13^C_3_,^15^N]l-Serine uptake assay

A total of 30,000 cells per well were typically plated in 24-well plates and following 72 h of gene silencing, the assay medium was renewed. For overexpression studies, following 24 h of transfection, 80,000 cells were typically plated in 24-well plates for 24 h and the assay medium was renewed afterwards. After 120 min from renewal of the medium, cells were supplemented with 100 μM [^13^C_3_,^15^N]l-serine (Cambridge Isotope Laboratories; CNLM-474-H-PK) in PBS for 1, 3 and 6 min. In experiment for Fig. 5b and Extended Data Fig. 4a, cells were provided with a mix containing the following labeled amino acids at 100 μM in PBS: [^13^C_3_,^15^N]l-serine, [^13^C_5_,^15^N]l-methionine, [^13^C_4_,^15^N]l-aspartate, [^13^C_5_,^15^N]l-glutamate, [^13^C_11_,^15^N_2_]l-tryptophan, [^13^C_5_,^15^N]l-valine, [^13^C_6_,^15^N]l-isoleucine, [^13^C_6_,^15^N]l-leucine, [^13^C_9_,^15^N]l-phenylalanine (Cambridge Isotope Laboratories; MSK-CAA-1) for 1, 3 and 6 min. Regarding Extended Data Fig. 4h, 300,000 HCT116 stably expressing EGFPN1–SLC6A14 were plated in six-well plates. After 24 h the medium was renewed with assay medium and after 2 h cells were supplemented with 200 μM [^13^C_3_,^15^N]l-serine (Cambridge Isotope Laboratories; CNLM-474-H-PK) and 0–400 μM [^13^C_6_,^15^N]l-leucine (Cambridge Isotope Laboratories; CNLM-281-H-PK) in PBS for 1, 3 and 6 min. Plates were washed with ice-cold PBS and metabolites were extracted with ice-cold extraction buffer consisting of methanol/acetonitrile/H_2_O (50:30:20; all LC–MS grade, Fisher Scientific). Extraction buffer volume per well was adjusted to cell number, using cell-count numbers from matching plates. Lysates were transferred to 1.5-ml Eppendorf tubes on ice, vortexed for 30 s and then centrifuged at 18,000*g* for 10 min at 4 °C. Supernatants were collected and stored at −80 °C for subsequent LC–MS analysis.

### [^13^C_3_,^15^N]l-Serine consumption assay

A total of 7,000 cells per well were typically plated in 96-well plates in assay medium and following 72 h of gene silencing. For overexpression studies, following 24 h of transfection, 20,000 cells were typically plated in 96-well plates for 24 h. Assay medium was then renewed (*t* = 0) containing 50 μM [^13^C_3_,^15^N]l-serine (Cambridge Isotope Laboratories; CNLM-474-H-PK). After 8 h (*t* = 8 h) 10 μl medium per well was lysed in 240 μl with ice-cold extraction buffer consisting of methanol/acetonitrile/H_2_O (50:30:20; all LC–MS grade, Fisher Scientific). Lysates were vortexed for 30 s and then centrifuged at 18,000*g* for 10 min at 4 °C. Supernatants were collected and stored at −80 °C for subsequent LC–MS analysis. Cell numbers were counted at *t* = 0 and at *t* = 8 h using a CellDrop FL automated cell counter (Cambridge Bioscience; DeNovix). Serial dilutions of [^13^C_3_,^15^N]l-serine starting from 100 μM were also analyzed using LC–MS to generate [^13^C_3_,^15^N]l-serine standard curve for each experiment. We calculated [^13^C_3_,^15^N]l-serine consumption (fmol per cell h^−^^1^) using [^13^C_3_,^15^N]l-serine concentration and cell number difference between *t* = 0 and *t* = 8 h.

### Amino acid consumption assays

For Fig. 1c,e,h, 7,000 cells per well were typically plated in 96-well plates in assay medium and following 72 h of RNAi-mediated gene silencing, 5 μl medium per well were lysed in 245 μl with ice-cold extraction buffer consisting of methanol/acetonitrile/H_2_O (50:30:20; all LC–MS grade, Fisher Scientific). Lysates were vortexed for 30 s and then centrifuged at 18,000*g* for 10 min at 4 °C. Supernatants were collected and stored at −80 °C for subsequent LC–MS analysis.

For Figs. 3i, 4h and 5a, 8,000 cells per well were typically plated in 96-well plates in assay medium and following 48 h of RNAi-mediated gene silencing, assay medium were renewed (0.2 ml medium per well). The medium samples were acquired at *t* = 0 h and at *t* = 24 h. Then, 50 μl medium per well were extracted in 50 μl chloroform (HPLC grade, Thermo Fisher Scientific), 150 μl methanol (OPTIMA, LC–MS grade, Thermo Fisher Scientific) and 100 μl H_2_O (OPTIMA, LC–MS grade, Thermo Fisher Scientific) containing 1 nmol *scyllo*-Inositol (Merck, I8132) and 1 nmol l-norleucine (Merck, N8513) (internal standards). All lysates were vortexed and centrifuged at 14,800*g* for 10 min at 4 °C. The aqueous phase was transferred to a fresh Eppendorf extraction tube, dried using centrifugal evaporation under vacuum (SpeedVac, RVC 2-33 CDplus, Martin Christ Gefriertrocknungsanlagen), derivatized and analyzed using GC–MS as described below. For each identified polar amino acid, total pmol per well at *t* = 24 h were subtracted from total pmol per well at *t* = 0 h and divided by respective cell numbers, providing pmol consumed/secreted over a period of 24 h.

### [U-^13^C_6_]d-Glucose tracing

A total of 300,000 cells were plated in six-well plates and left to adhere overnight. Cells were washed with PBS and the medium was replaced with SILAC DMEM Flex Medium, no glucose, no phenol red (A2493901, Thermo Fisher Scientific) containing [U-^13^C_6_]d-glucose, 99% (Cambridge Isotope Laboratories/CK Isotopes) concentration. After 60-min and 180-min incubation periods, metabolites were extracted with ice-cold extraction buffer, consisting of methanol/acetonitrile/H_2_O (50:30:20; all LC–MS grade, Fisher Scientific). Lysis buffer volume added per well was adjusted to cell number, using cell-count numbers from matching plates.

### [^13^C_3_,^15^N]l-Serine mitochondrial uptake assays

For the RNAi-mediated silencing experiments, 10^6^ HCT116 cells cultured in 10-cm dishes and after 24 h, cells were transfected with NTC siRNA (control) or siRNA against *SLC25A15* using the Lullaby transfection reagent (OZ Biosciences, LL70500) according to the manufacturer’s instructions. After 48 h cells received a second shot of siRNA against either NTC (control) or *SLC25A15*. After 24 h, the medium was renewed and cells were left for further 16 h to stabilize in assay medium as described above. Cells were then supplemented with [^13^C_3_,^15^N]l-serine (0.2 mM final; Cambridge Isotope Laboratories; CNLM-474-H-PK) for 60 min.

For the overexpression experiments, 4 × 10^6^ HCT116 cells (EV, control) or HCT116 cells overexpressing FLAG-tagged SLC25A15 were plated on 10-cm dishes and after 48 h, the medium was renewed and cells were left for further 16 h to stabilize in assay medium as described above. Cells were then supplemented with [^13^C_3_,^15^N]l-serine (0.2 mM final; Cambridge Isotope Laboratories; CNLM-474-H-PK) for 60 min.

Cytosol/mitochondrial compartmentalization was performed with a modified version of a previously published assay^70^. The medium was removed and cells were washed once with 0.9% NaCl in ddH_2_O solution. A solution of potassium phosphate monobasic (2 mM), potassium chloride (120 mM), HEPES (3 mM), EGTA (1 mM), BSA (3 g l^−^^1^) and digitonin (100 μg ml^−^^1^) at pH 7.2 was added to each plate (500 μl) and plates were incubated at 37 °C for 2 min. The solution (cytosol-enriched fraction) was removed and quenched with 500 μl methanol (OPTIMA, LC–MS grade, Thermo Fisher Scientific) on ice. Plates were then washed twice with 0.9% NaCl in ddH_2_O solution and mitochondria-enriched fractions were collected and quenched in 500 μl methanol (OPTIMA, LC–MS grade, Thermo Fisher Scientific) on ice. Total cell lysates were collected from plates just washed with saline solution. In all fractions 500 μl chloroform (HPLC grade, Thermo Fisher Scientific) were added, while 500 μl H_2_O was added to the mitochondrial-enriched and total cell extracts so that volumes were equal between all extracts. The methanol contained l-norleucine (Merck, N8513) and *scyllo*-Inositol (Merck, I8132) (1 nmol per sample) as internal standards. All lysates were vortexed and centrifuged at 14,800*g* for 10 min at 4 °C. The aqueous phase was transferred to a fresh Eppendorf extraction tube, dried using centrifugal evaporation under vacuum (SpeedVac, RVC 2-33 CDplus, Martin Christ Gefriertrocknungsanlagen), derivatized and analyzed using GC–MS as described below.

For Extended Data Fig. 3m, following 24 h of DNA plasmid transfection as described above, 400,000 HEK293 cells were typically plated in 6-cm tissue culture plates for 24 h and the assay medium was renewed afterwards. After 120 min of medium equilibration, cells were supplemented with 100 μM [^13^C_3_,^15^N]l-serine (Cambridge Isotope Laboratories; CNLM-474-H-PK) in PBS for 60 min. Rapid subcellular fractionation followed by LC–MS was performed based on a previously reported protocol^39^. Plates were washed twice with ice-cold PBS and cells were collected into Eppendorf tubes in 1 ml ice-cold PBS. Following centrifugation at 13,500*g* for 10 s (4 °C), the supernatant was removed and the cell pellet was resuspended in 1 ml ice-cold PBS containing 1 mg ml^−1^ digitonin (D141; Sigma-Aldrich, Merck). Following centrifugation at 13,500*g* for 10 s (4 °C) the supernatant (‘cytosolic fraction’) and the pellet (‘mitochondrial-enriched fraction’) were collected. Metabolites were extracted with 4 ml ice-cold 62.5:37.5 (*v*/*v*) methanol/acetonitrile (‘cytosolic fraction’) or 100 µl 50:30:20 (*v*/*v*/*v*) methanol/acetonitrile/H_2_O (‘mitochondrial-enriched fraction’). Lysates were transferred to 1.5-ml Eppendorf tubes on ice, vortexed for 30 s and then centrifuged at 18,000*g* for 10 min at 4 °C. Supernatants were collected and stored at −80 °C for subsequent LC–MS analysis.

### Amino acid quantification in tumors and serum

Tumor tissue samples were snap-frozen and stored at −80 °C. Tumor samples were prepared in cold (−20 °C) solvent consisting of methanol:acetonitrile:H_2_O (50:30:20). Then, 1 ml solvent was used per 20 mg of tissue and samples were homogenized using a TissueLyser II (QIAGEN). Lysates were centrifuged at 18,000*g* for 10 min at 4 °C. Supernatants were collected and stored at −80 °C for subsequent LC–MS analysis. Serum was isolated from terminal bleeds and stored at −80 °C. Then, 5 μl serum was extracted with 15 μl methanol (OPTIMA, LC–MS grade, Thermo Fisher Scientific) for 5 min on ice and centrifuged for 10 min at 4 °C and 18,000*g*. Samples were dried using centrifugal evaporation under vacuum (SpeedVac, RVC 2-33 Cdplus, Martin Christ Gefriertrocknungsanlagen) and partitioned in 50 μl chloroform, 150 μl methanol and 150 μl H_2_O buffer. Samples were centrifuged for 10 min at 4 °C and 18,000*g*. The polar phase was then dried using centrifugal evaporation under vacuum (SpeedVac, RVC 2-33 CDplus, Martin Christ Gefriertrocknungsanlagen) and was resuspended in 100 μl methanol:H_2_O (1:1) (OPTIMA, LC–MS grade, Thermo Fisher Scientific) buffer. Samples were analyzed using LC–MS.

### LC–MS

UHPLC of the samples was performed using an Accela 600 LC system (Thermo Fisher Scientific). Metabolites were separated using a SeQuant ZIC-HILIC column (4.6 mm × 150 mm, 3.5 μm) or a SeQuant ZIC-pHILIC column (4.6 mm × 150 mm, 5 μm) (Merck). For HILIC separation, the mobile phase consisted of formic acid in water 0.1% (*v*/*v*) (A) and formic acid in acetonitrile 0.1% (*v*/*v*) (B) and a gradient program was used consisting of the following steps: linear increase of A from 30% to 70% between 0–2 min, 92% A 12–14 min and linear decrease of A to 30% at 15–20 min. For the separation in pHILIC, the mobile phase consisted of an aqueous solution of ammonium carbonate (20 mM, pH 9.2) (A) and acetonitrile (B) and a gradient program was used consisting of the following steps: linear increase of A from 20% to 80% between 0–30 min, 92% A 31–37 min and linear decrease of A to 20% at 37–46 min. The flow rate was kept at 0.3 ml min^−^^1^ and column temperature was maintained at 28 °C. An Exactive (Orbitrap) mass spectrometer (Thermo Fisher Scientific) was operated in both positive and negative electrospray ionization (ESI) modes. The instrument was operated in full scan mode over a mass range of 70–1,200 *m*/*z* at a resolution of 50,000. Data were recorded using Xcalibur v.2.2 software (Thermo Fisher). The capillary temperature was 320 °C and the sheath and auxiliary gas flow rates were 50 and 17 units, respectively.

For Fig. 5i and Extended Data Fig. 4g, a ZIC-pHILIC column (SeQuant; 150 mm × 2.1 mm, 5 µm; Merck) coupled with a ZIC-pHILIC guard column (SeQuant; 20 mm × 2.1 mm) using an Ultimate 3000 HPLC system (Thermo Fisher Scientific) was used as previously described^71^. Chromatographic separation was performed using a 15-min linear gradient starting with 20% ammonium carbonate (20 mM, pH 9.2) and 80% acetonitrile, terminating at 20% acetonitrile at a constant flow rate of 200 µl min^–1^. The column temperature was held at 45 °C. A Q-Exactive Orbitrap mass spectrometer (Thermo Fisher Scientific) equipped with ESI was coupled to the HPLC system with a polarity switching mode with a resolution (RES) of 70,000 at 200 *m*/*z* to enable both positive and negative ions to be detected across a mass range of 75 to 1,000 *m*/*z* (automatic gain control target of 1 × 10^6^ and maximal injection time of 250 ms).

LC–MS analysis was performed as previously described^72^. The LC–MS raw data files were converted into mzML files using ProteoWizard and imported to MZMine 2.53 for peak extraction and sample alignment. Metabolite identification was performed by matching exact *m*/*z* values (±5 ppm) and retention times (±8%) of standards in an in-house-made database. For isotope-tracing experiments, an in-house-made database including all possible ^13^C and ^15^N isotopic *m*/*z* values of the relevant metabolites was used for the assignment of LC–MS signals. Resultant peak lists were exported as .csv files and used for plotting and analysis.

For Fig. 7h–k and Extended Data Figs. 4h and 6f–i, Metabolite analysis was performed by LC–MS using a Q-Exactive Plus (Orbitrap) mass spectrometer (Thermo Fisher) coupled with a Vanquish UHPLC system (Thermo Fisher) as described previously^73^. Chromatographic separation was performed on a SeQuant ZIC-pHILIC (Merck) column (5-μm particle size, polymeric, 150 × 4.6 mm). The injection volume was 5 μl, the oven temperature was maintained at 25 °C and the autosampler tray temperature was maintained at 4 °C. Chromatographic separation was achieved using a gradient program at a constant flow rate of 300 μl min^−1^ over a total run time of 25 min. The elution gradient was programmed as decreasing percentage of B from 80% to 5% during 17 min, holding at 5% of B during 3 min and finally re-equilibrating the column at 80% of B during 4 min. Solvent A was 20 mM ammonium carbonate solution in water supplemented by 1.4 ml l^−1^ of a solution of ammonium hydroxide at 35% in water and solvent B was acetonitrile. MS was performed with positive/negative polarity switching using a Q-Exactive Plus Orbitrap (Thermo Fisher) with a HESI II probe. MS parameters were as follows: spray voltage 3.5 and 3.2 kV for positive and negative modes, respectively; probe temperature 320 °C; sheath and auxiliary gases were 30 and 5 arbitrary units, respectively; and full scan range of 65–975 *m*/*z* with settings of automatic gain control target and resolution as balanced and high (1 × 10^6^ and 70,000), respectively. Data were recorded using Xcalibur 4.2.47 software (Thermo Fisher). Mass calibration was performed for both ESI polarities before analysis using the standard Thermo Fisher Calmix solution. To enhance calibration stability, lock-mass correction was also applied to each analytical run using ubiquitous low-mass contaminants. Parallel reaction monitoring acquisition parameters were a resolution of 17,500 and collision energies set individually in high-energy collisional dissociation mode. Metabolites were identified and quantified by accurate mass and retention time and by comparison to the retention times, mass spectra and responses of known amounts of authentic standards using TraceFinder 4.1 EFS software (Thermo Fisher). Samples were supplemented with [^13^C_5_,^15^N]l-valine (Cambridge Isotope Laboratories; CNLM-442-H-0.25) (5 μM) during the extraction process and relative abundances were calculated over the peak area of this internal standard.

### GC–MS

Dried samples were washed three times with methanol and derivatized first with 20 μl methoxyamine hydrochloride (Merck, 89803) solution in pyridine (Merck, 270970) (20 mg ml^−^^1^) for 16 h and then with 20 μl BSTFA + 1% TMCS silylation reagent (Thermo Fisher Scientific, TS-38831) for 30 min. Metabolite analysis was performed by GC–MS using an Agilent 7890B-7000C GC-triple-quadrupole MS. Splitless injection (injection temperature 250 °C) onto a 30 m + 10 m × 0.25 mm DB-5MS + DG column (Agilent J&W) was used, using helium as the carrier gas, in electron ionization mode. The initial oven temperature was 70 °C (2 min), followed by temperature gradients to 295 °C at 12.5 °C min^−1^, then to 320 °C at 25 °C min^−1^ (held for 3 min). Data analysis was performed using our in-house-developed software MANIC (v.3.0), based on the software package GAVIN^74^. Label incorporation was calculated by subtracting the natural abundance of stable isotopes from the observed amounts. Metabolites were identified and quantified in comparison to authentic standards and *scyllo*-Inositol as an internal standard (Sigma, I8132).

### Gene expression and overall survival profiling

Expression and overall survival data of all genes of interest in colorectal, breast and pancreatic cancer primary tumors and metastases as well as expression data for healthy samples were obtained from TCGA, TARGET and GTEx databases and values were plotted as log_2_ (TPM + 0.001). Expression data from the panels of breast and colorectal cancer cell lines were obtained from the Cancer Cell Line Encyclopedia database and plotted as log_2_(RPKM + 1). All data were generated using the UCSC Xena Brower (xena.ucsc.edu/)^75^ and analyzed and plotted using Rstudio (v.1.4.1717) or Prism 9 (v.9.3.1; GraphPad Software).

### Subcutaneous xenografts

All in vivo work was carried out in compliance with the Animals (Scientific Procedures) Act 1986 and the EU Directive 2010 (PPLs 70/8645 and PP6345023) and was sanctioned by the local animal welfare ethical review board (University of Glasgow). Then, 3 × 10^6^ cells HCT116 cells were resuspended in 100 μl PBS and introduced into each mouse (CD-1 Nude females, 7–8 weeks old; Charles River Laboratories) by subcutaneous injection on the flank. Tumors were measured using calipers three times weekly by staff blinded to the experimental outcome. Tumor volume (*V*) was calculated using the formula *V* = (length × width^2^)/2. Upon tumor formation (*V* ~70 mm^3^) mice were assigned into no-DOX (control) and DOX (test condition) regimes in a manner ensuring a consistent average starting volume across the groups. DOX was given by oral gavage for 5 d (dissolved in sterile water, 2 mg d^−1^) and then in drinking water (0.2 mg ml^−1^ dissolved in a 5 mg ml^−1^ sucrose solution, changed twice per week). Tumor growth was monitored until the tumor-related end point was reached (tumors measuring 14–15 mm in any dimension or ulcerated) and animals humanely culled using Schedule 1 methods. Tumor-related end points were not exceeded in any animals involved in this study. Each mouse was an experimental unit housed in cages of *n* = 5 where DOX and no-DOX were housed in separate cages. A total of 67 mice were injected with cells (21 NTC, 24 SLC6A14/12A4 and 22 SLC6A14/25A15). Before enrollment, four mice were excluded from the study as they either failed to develop tumors (one NTC and one SLC6A14/12A4) or had substantially delayed tumor growth/regressed (two SLC6A14/25A15).

### Statistics and reproducibility

All datasets were analyzed and plotted using Prism 9 (v.9.3.1; GraphPad Software) unless otherwise stated. Differences between groups were tested for normal distribution and analyzed using the appropriate statistical test, as mentioned in each figure legend. Error bars represent s.d. unless otherwise stated. Biological replicates are cells grown in separate plates/wells in which experimental conditions were replicated within an experiment and each well yielded a sample that was analyzed (for example by LC–MS) independently. The heat map in Fig. 1c was generated using the ComplexHeatmap tool^76^ in Rstudio (v.1.4.1717). No statistical methods were used to predetermine sample sizes but our sample sizes are similar to those reported previously^8,9,13,72,77^. Animals were assigned to treatment groups in a manner ensuring a consistent average starting volume across the groups. The RNAi screen was performed by an independent investigator blinded to the experimental conditions. Data collection and analysis for the in vivo work was performed by an independent investigator blinded to the conditions of the experiments. LC–MS samples were analyzed by independent investigators blinded to the experimental conditions/treatments. For all other work, data collection and analysis were not performed blinded to the conditions of the experiments.

### Reporting summary

Further information on research design is available in the Nature Portfolio Reporting Summary linked to this article.

### Supplementary information

Supplementary Information Supplementary Tables 1–5.

Reporting Summary

### Source data

Source Data Table 1 Statistical source data related to Fig. 1.

Source Data Table 2 Statistical source data related to Fig. 2.

Source Data Table 3 Statistical source data related to Fig. 3.

Source Data Table 4 Statistical source data related to Fig. 4.

Source Data Table 5 Statistical source data related to Fig. 5.

Source Data Table 6 Statistical source data related to Fig. 6.

Source Data Table 7 Statistical source data related to Fig. 7.

Source Data Extended Data Table 1 Statistical source data related to Extended Data Fig. 1.

Source Data Extended Data Table 2 Statistical source data related to Extended Data Fig. 2.

Source Data Extended Data Table 3 Statistical source data related to Extended Data Fig. 3.

Source Data Extended Data Table 4 Statistical source data related to Extended Data Fig. 4.

Source Data Extended Data Table 5 Statistical source data related to Extended Data Fig. 5.

Source Data Extended Data Table 6 Statistical source data related to Extended Data Fig. 6.

Source Data Extended Data Table 7 Statistical source data related to Extended Data Fig. 7.

Source Data Fig. 1 Unprocessed blots.

Source Data Fig. 2 Unprocessed blots.

## Data Availability

Associated raw data are provided as Source Data Files associated with each main or extended data figure. Original datasets, analyses and methodological details are available from the source data supplementary files and publicly available from researchdata.gla.ac.uk/. Information regarding experimental design and reagents can also be found in the Reporting Summary. Source data are provided with this paper.
