# Supplementary material for: Phenotypic profiling of solute carriers characterizes serine transport in cancer
Source: Nat Metab. 2023 Dec 8;5(12):2148–68. doi: 10.1038/s42255-023-00936-2 (PMC10730406; doi:10.1038/s42255-023-00936-2)
Supplement: Supplementary file 1 — Supplementary Tables 1–5. [file 42255_2023_936_MOESM1_ESM.pdf]

nature metabolism

Article https://doi.org/10.1038/s42255-023-00936-2

Phenotypic profiling of solute carriers characterizes serine transport in cancer

In the format provided by the authors and unedited

Supplementary Table 1 - Reported SLCs		
SLC	Reported Serine and/or Glycine Transport	Reference
SLC1A4	Serine	https://www.ncbi.nlm.nih.gov/pmc/articles/PMC6156681/
SLC1A5	Serine	https://pubmed.ncbi.nlm.nih.gov/27272177/
SLC38A5	Serine	https://pubmed.ncbi.nlm.nih.gov/11698233/
SLC38A7	Serine	https://link.springer.com/content/pdf/10.1007/s00424-013-1393-y.pdf
SLC6A9	Glycine	https://pubmed.ncbi.nlm.nih.gov/27481395/
SLC32A1	Glycine	https://pubmed.ncbi.nlm.nih.gov/19843525/
SLC36A1	Glycine	https://www.ncbi.nlm.nih.gov/pmc/articles/PMC2342508/
SLC6A14	Serine and Glycine	https://www.ncbi.nlm.nih.gov/pmc/articles/PMC4638383/
SLC7A8	Serine and Glycine	https://www.ncbi.nlm.nih.gov/pmc/articles/PMC7182634/#CR6
SLC38A1	Serine and Glycine	https://link.springer.com/content/pdf/10.1007/s00424-013-1393-y.pdf
SLC38A2	Serine and Glycine	https://link.springer.com/content/pdf/10.1007/s00424-013-1393-y.pdf

Supplementary Table 2 - Oligos						
ID	Target	Species	Sequence	Supplier	Catalogue No	Application
NTC1	ON-TARGETplus Non-targeting Control	H. sapiens	UGGUUUACAUGUCGACUAA	Dharmacon; Horizon Discovery	D-001810-01	RNAi
NTC2	ON-TARGETplus Non-targeting Control	H. sapiens	UGGUUUACAUGUUGUGUGA	Dharmacon; Horizon Discovery	D-001810-02	RNAi
NTC3	ON-TARGETplus Non-targeting Control	H. sapiens	UGGUUUACAUGUUUUCUGA	Dharmacon; Horizon Discovery	D-001810-03	RNAi
NTC4	ON-TARGETplus Non-targeting Control	H. sapiens	UGGUUUACAUGUUUUCCUA	Dharmacon; Horizon Discovery	D-001810-04	RNAi
SLC1A5_si01	SLC1A5	H. sapiens	GCCUUUCGCUCAUACUCUA	Dharmacon; Horizon Discovery	J-007429-05	RNAi
SLC1A5_si02	SLC1A5	H. sapiens	GGUCGACCAUAUCUCCUUG	Dharmacon; Horizon Discovery	J-007429-06	RNAi
SLC1A5_si03	SLC1A5	H. sapiens	GCAAGGAGGUGCUCGAUUC	Dharmacon; Horizon Discovery	J-007429-07	RNAi
SLC1A5_si04	SLC1A5	H. sapiens	UGAUACAAGUGAAGAGUGA	Dharmacon; Horizon Discovery	J-007429-08	RNAi
SLC38A5_si01	SLC38A5	H. sapiens	CGGGAUAUCUUUGGAGUUA	Dharmacon; Horizon Discovery	J-007562-17	RNAi
SLC38A5_si02	SLC38A5	H. sapiens	UCAUGGAUUUCGAGGGGAA	Dharmacon; Horizon Discovery	J-007562-18	RNAi
SLC38A5_si03	SLC38A5	H. sapiens	AUAAAGAUGUUGAGCUACC	Dharmacon; Horizon Discovery	J-007562-19	RNAi
SLC38A5_si04	SLC38A5	H. sapiens	GCAGUGUGAAGGCGGAGAU	Dharmacon; Horizon Discovery	J-007562-20	RNAi
SLC1A4_si01	SLC1A4	H. sapiens	GUACGUAUGCAACCGAUUA	Dharmacon; Horizon Discovery	J-007428-09	RNAi
SLC1A4_si02	SLC1A4	H. sapiens	UCACAAAGCCAUAAAGCGU	Dharmacon; Horizon Discovery	J-007428-10	RNAi
SLC1A4_si03	SLC1A4	H. sapiens	GGGAAAUACAUCUUCGCAU	Dharmacon; Horizon Discovery	J-007428-11	RNAi
SLC1A4_si04	SLC1A4	H. sapiens	AGAAAUGAAUACCGUGUUU	Dharmacon; Horizon Discovery	J-007428-12	RNAi
SLC38A7_si01	SLC38A7	H. sapiens	CCAUCUUCAUCGUCGUCAA	Dharmacon; Horizon Discovery	J-007328-18	RNAi
SLC38A7_si02	SLC38A7	H. sapiens	CAUUCUUAGUCCACGAUCA	Dharmacon; Horizon Discovery	J-007328-19	RNAi
SLC38A7_si03	SLC38A7	H. sapiens	GGGUGAAGAGAGUCGGUAG	Dharmacon; Horizon Discovery	J-007328-20	RNAi
SLC38A7_si04	SLC38A7	H. sapiens	CUUGGUACACAGACCGCAA	Dharmacon; Horizon Discovery	J-007328-21	RNAi
SLC6A9_si01	SLC6A9	H. sapiens	GGGCAACCAGAUCGAGUUU	Dharmacon; Horizon Discovery	J-007609-09	RNAi
SLC6A9_si02	SLC6A9	H. sapiens	CUCCAGGACUCCCGGAUAU	Dharmacon; Horizon Discovery	J-007609-10	RNAi
SLC6A9_si03	SLC6A9	H. sapiens	UCAUGUACAUCUACGGGCA	Dharmacon; Horizon Discovery	J-007609-11	RNAi
SLC6A9_si04	SLC6A9	H. sapiens	GGAGUGGGCUAUGGUAUGA	Dharmacon; Horizon Discovery	J-007609-12	RNAi
SLC36A1_si01	SLC36A1	H. sapiens	CUGAGUGAGAGGCGUAUAA	Dharmacon; Horizon Discovery	J-007550-09	RNAi
SLC36A1_si02	SLC36A1	H. sapiens	GGAAUGAAGACUACCACGA	Dharmacon; Horizon Discovery	J-007550-10	RNAi
SLC36A1_si03	SLC36A1	H. sapiens	CUUCUUUGGCACAGCGAUU	Dharmacon; Horizon Discovery	J-007550-11	RNAi
SLC36A1_si04	SLC36A1	H. sapiens	GCUUCUGUGUUUCGCCAAA	Dharmacon; Horizon Discovery	J-007550-12	RNAi
SLC6A14_si01	SLC6A14	H. sapiens	GGAAUAAAGUGGCGCUCCA	Dharmacon; Horizon Discovery	J-007601-09	RNAi
SLC6A14_si02	SLC6A14	H. sapiens	ACGCAGUGGGAUUAGGAAA	Dharmacon; Horizon Discovery	J-007601-10	RNAi
SLC6A14_si03	SLC6A14	H. sapiens	GUUUCAAGGUGUGGGAAUU	Dharmacon; Horizon Discovery	J-007601-11	RNAi
SLC6A14_si04	SLC6A14	H. sapiens	CGUCUGGCAAGGUGGUAUA	Dharmacon; Horizon Discovery	J-007601-12	RNAi
SLC38A2_si01	SLC38A2	H. sapiens	CUGAAAGACCGCAGCCGUA	Dharmacon; Horizon Discovery	J-007559-09	RNAi
SLC38A2_si02	SLC38A2	H. sapiens	GCACAUUUAUCGUACGUCA	Dharmacon; Horizon Discovery	J-007559-10	RNAi
SLC38A2_si03	SLC38A2	H. sapiens	CAAUUGGGAUAUAAGGCAU	Dharmacon; Horizon Discovery	J-007559-11	RNAi
SLC38A2_si04	SLC38A2	H. sapiens	GGGAAUACCAAGAGUUGUU	Dharmacon; Horizon Discovery	J-007559-12	RNAi
SLC38A1_si01	SLC38A1	H. sapiens	CAAAAGUGGACUCGAAUUA	Dharmacon; Horizon Discovery	J-007558-10	RNAi
SLC38A1_si02	SLC38A1	H. sapiens	CCACAGGGAAGUUCGUAAU	Dharmacon; Horizon Discovery	J-007558-11	RNAi
SLC38A1_si03	SLC38A1	H. sapiens	GAGAAUCAUGUGUACCUAA	Dharmacon; Horizon Discovery	J-007558-12	RNAi
SLC38A1_si04	SLC38A1	H. sapiens	GAUAGUUACCUUUGGCAUA	Dharmacon; Horizon Discovery	J-007558-13	RNAi
SLC25A28_si01	SLC25A28	H. sapiens	GUGUAUGAGUUCUUCAAAU	Dharmacon; Horizon Discovery	J-007368-17	RNAi
SLC25A28_si02	SLC25A28	H. sapiens	GUGAAGUAGCACUGAACGA	Dharmacon; Horizon Discovery	J-007368-18	RNAi
SLC25A28_si03	SLC25A28	H. sapiens	CCAUGAACCCUGCGGAAGU	Dharmacon; Horizon Discovery	J-007368-19	RNAi
SLC25A28_si04	SLC25A28	H. sapiens	CUUCAGGACGGUAUAUCAA	Dharmacon; Horizon Discovery	J-007368-20	RNAi
SLC25A15_si01	SLC25A15	H. sapiens	GCCUCUGGCUUGCGGUAUA	Dharmacon; Horizon Discovery	J-007474-06	RNAi
SLC25A15_si02	SLC25A15	H. sapiens	GCACUAAUCGCCAACAUCG	Dharmacon; Horizon Discovery	J-007474-07	RNAi
SLC25A15_si03	SLC25A15	H. sapiens	GAAUUAGGCCCUGUACCUU	Dharmacon; Horizon Discovery	J-007474-08	RNAi
SLC25A15_si04	SLC25A15	H. sapiens	CAGGCAGGAUUUAUCAGAA	Dharmacon; Horizon Discovery	J-007474-09	RNAi
SLC7A8_si01	SLC7A8	H. sapiens	CCACGAAGGACAAGGACGU	Dharmacon; Horizon Discovery	J-007618-09	RNAi
SLC7A8_si02	SLC7A8	H. sapiens	UGGCCAUGAUCCACGUGAA	Dharmacon; Horizon Discovery	J-007618-10	RNAi
SLC7A8_si03	SLC7A8	H. sapiens	AGAUGUGUGUGGUCGUGUA	Dharmacon; Horizon Discovery	J-007618-11	RNAi
SLC7A8_si04	SLC7A8	H. sapiens	CCCAAGUGUUUCAGUGACU	Dharmacon; Horizon Discovery	J-007618-12	RNAi
SLC12A4_si01	SLC12A4	H. sapiens	GGGAACAACAUUCGCAGCA	Dharmacon; Horizon Discovery	J-007385-09	RNAi
SLC12A4_si02	SLC12A4	H. sapiens	ACAGUGAGGGCGACGAGAA	Dharmacon; Horizon Discovery	J-007385-10	RNAi
SLC12A4_si03	SLC12A4	H. sapiens	UGGAUGACUCGGACGGACA	Dharmacon; Horizon Discovery	J-007385-11	RNAi
SLC12A4_si04	SLC12A4	H. sapiens	CCACCAAGCUGAGCCGAAU	Dharmacon; Horizon Discovery	J-007385-12	RNAi
SLC25A1_si01	SLC25A1	H. sapiens	GCGCACAAAUACCGGAACA	Dharmacon; Horizon Discovery	J-007468-07	RNAi
SLC25A1_si02	SLC25A1	H. sapiens	GGAAGACGGACUAAGCCUA	Dharmacon; Horizon Discovery	J-007468-08	RNAi
SLC25A1_si03	SLC25A1	H. sapiens	GCCAUCCGCUUCUUCGUCA	Dharmacon; Horizon Discovery	J-007468-09	RNAi
SLC25A1_si04	SLC25A1	H. sapiens	GGUUUGGAAUGUUCGAGUU	Dharmacon; Horizon Discovery	J-007468-10	RNAi
PHGDH_CRISPR-1	PHGDH	H. sapiens	TGCAAGATCTTCCGGCAGCA	IDT	n/a	CRISPR
PHGDH_CRISPR-2	PHGDH	H. sapiens	TGCCGGAAGATCTTGCAAGA	IDT	n/a	CRISPR
NTC_gRNA_A	NTC	H. sapiens	AAAATAGCAGTAAACTCAAC	IDT	n/a	CRISPR
NTC_gRNA_B	NTC	H. sapiens	GAAGAAGAATTGGGGATGATG	IDT	n/a	CRISPR
SLC6A14_CRISPR-1	SLC6A14	H. sapiens	TCAGTAAAGTGGCGCTCCAA	IDT	n/a	CRISPR
SLC12A4_gRNA	SLC12A4	H. sapiens	AGAGCTGGACATCCGCCCAA	IDT	n/a	CRISPR
SLC25A15_gRNA_1	SLC25A15	H. sapiens	GGCTTCCGTGGCTTCTACAA	ThermoFisher Scientific	n/a	CRISPR
SLC25A15_gRNA_2	SLC25A15	H. sapiens	GGCACTTCACGAGCTCCGTG	ThermoFisher Scientific	n/a	CRISPR

Supplementary Table 3 - DNA constructs			
Name	Backbone	Source	Ref
pDONR221_SLC12A4	pDONR221	RESOLUTE Consortium, Giulio Superti-Furga	Addgene; 131932
pDONR221_SLC16A5	pDONR221	RESOLUTE Consortium, Giulio Superti-Furga	Addgene; 131923
pDONR221_SLC25A15	pDONR221	RESOLUTE Consortium, Giulio Superti-Furga	Addgene; 131967
pDONR221_SLC24A1	pDONR221	RESOLUTE Consortium, Giulio Superti-Furga	Addgene; 132049
pDONR221_SLC35C2	pDONR221	RESOLUTE Consortium, Giulio Superti-Furga	Addgene; 132146
pDONR221_SLC6A14	pDONR221	RESOLUTE Consortium, Giulio Superti-Furga	Addgene; 131865
pDONR221_SLC38A2	pDONR221	RESOLUTE Consortium, Giulio Superti-Furga	Addgene; 132059
pDONR221_SLC38A5	pDONR221	RESOLUTE Consortium, Giulio Superti-Furga	Addgene; 132118
pDONR221_SLC1A5	pDONR221	RESOLUTE Consortium, Giulio Superti-Furga	Addgene; 131974
pEGFP-C1	pEGFP-C1	Host lab	n/a
pEGFP-N1	pEGFP-N1	Host lab	n/a
pcDNA3.1 (+)	pcDNA3.1 (+)	Host lab	n/a
pcDNA3.1 (+) FLAG	pcDNA3.1 (+)	This study	n/a
SLC6A14 pEGFPN1	pEGFP-N1	This study	n/a
SLC6A14; V128E pEGFPN1	pEGFP-N1	This study	n/a
SLC6A14; V128G pEGFPN1	pEGFP-N1	This study	n/a
SLC6A14; V128W pEGFPN1	pEGFP-N1	This study	n/a
SLC6A14_FLAG pcDNA3.1(+)	pcDNA3.1 (+)	This study	n/a
SLC25A15_FLAG pcDNA3.1(+)	pcDNA3.1 (+)	This study	n/a
SLC38A2 pEGFPN1	pEGFP-N1	This study	n/a
SLC38A2_FLAG pcDNA3.1(+)	pcDNA3.1 (+)	This study	n/a
SLC12A4 pEGFPN1	pEGFP-N1	This study	n/a
SLC12A4_FLAG pcDNA3.1(+)	pcDNA3.1 (+)	This study	n/a
SLC38A5 pEGFPN1	pEGFP-N1	This study	n/a
SLC38A5_FLAG pcDNA3.1(+)	pcDNA3.1 (+)	This study	n/a
SLC1A5 pEGFPN1	pEGFP-N1	This study	n/a
SLC1A5 pcDNA3.1(+) FLAG	pcDNA3.1 (+)	This study	n/a
pSpCas9 (BB) - 2A - Puro - (PX45Q) V2.0	PX459	Feng Zhang	Addgene; 62988
pSpCas9 (BB) - 2A - Puro_NTC	pSpCas9 (BB) - 2A - Puro - (PX45Q) V2.0	This study	n/a
Lenti-iCas9-neo	pInducer-20	Qin Yan	Addgene; 85400
Lenti-multi-Guide	pLentiGuide	Qin Yan	Addgene; 85401
pVSV-G	pCMV	Host lab	n/a
psPAX2	psPAX2	Host lab	n/a
Lenti-multi-Guide (LMG)_NTC gRNA1/2	Lenti-multi-Guide	This study	n/a
Lenti-multi-Guide (LMG)_SLC6A14/SLC12A4	Lenti-multi-Guide	This study	n/a
Lenti-multi-Guide (LMG)_SLC6A14/SLC25A15_gRNA_A	Lenti-multi-Guide	This study	n/a
Lenti-multi-Guide (LMG)_SLC6A14/SLC25A15_gRNA_B	Lenti-multi-Guide	This study	n/a

Supplementary Table 4 - qRT-PCR primers				
Target	ID	Species	Sequence	Supplier
GAPDH	GAPDH_Fw	H. sapiens	AGCCACATCGCTCAGACAC	IDT
GAPDH	GAPDH_Rv	H. sapiens	GCCCAATACGACCAAATC	IDT
Actin	Actin_Fw	H. sapiens	CCAACCGCGAGAAGATGA	IDT
Actin	Actin_Rv	H. sapiens	CCAGAGGCGTACAGGGATAG	IDT
SLC12A4	SLC12A4Fw_B	H. sapiens	GGCGCGGCGACTATGAC	IDT
SLC12A4	SLC12A4Rv_B	H. sapiens	GTGGTTGCCATGTCCGTC	IDT
SLC1A5	SLC1A5Fw	H. sapiens	CCTCCGCCGCCATCAAC	IDT
SLC1A5	SLC1A5Rv	H. sapiens	TGGAAGGGAAGATATTTCTCGCA	IDT
SLC38A5	SLC38A5Fw	H. sapiens	AACCTGAGTCTGAGTTGCGG	IDT
SLC38A5	SLC38A5Rv	H. sapiens	AGGGAGGGCTCCATTCATCT	IDT
SLC1A4	SLC1A4Fw	H. sapiens	AGACCCTTCAGTCCAGCGA	IDT
SLC1A4	SLC1A4Rv	H. sapiens	TGGAGGGAAACAGGTTTCTGG	IDT
SLC38A7	SLC38A7Fw	H. sapiens	GAAAGAATCCCCCAAGCTCCA	IDT
SLC38A7	SLC38A7Rv	H. sapiens	TGAGCAACACCCACCTGTTT	IDT
SLC6A9	SLC6A9Fw	H. sapiens	CTCTTCCCCAGAACAGAATGGT	IDT
SLC6A9	SLC6A9Rv	H. sapiens	CCCACGCTCGTCAGTACAA	IDT
SLC6A14	SLC6A14_Fw	H. sapiens	ATCGTCTGGCAAGGTGGTAT	IDT
SLC6A14	SLC6A14_Rv	H. sapiens	AATGCCTTTTGAAGCACCCT	IDT
SLC38A2	SLC38A2Fw	H. sapiens	AGCTGCTCTGAAAAGCCATTAT	IDT
SLC38A2	SLC38A2Rv	H. sapiens	CCAAAGGAAGTAGTACCTGGATG	IDT
SLC7A8	SLC7A8Fw	H. sapiens	CCTGTGGTATCATCGTAGGGAA	IDT
SLC7A8	SLC7A8Rv	H. sapiens	AAGCCCGTCACAATCCAGAC	IDT
SLC25A28	SLC25A28Fw	H. sapiens	CATATTGCCAATGGTGCGG	IDT
SLC25A28	SLC25A28Rv	H. sapiens	CCGGTGGTATGGTGAGTTGT	IDT
SLC25A15	SLC25A15Fw	H. sapiens	ACTAATCGCCAACATCGCTGA	IDT
SLC25A15	SLC25A15Rv	H. sapiens	CATTCTGCAGATCACTCAGCTT	IDT
SLC36A1	SLC36A1Fw	H. sapiens	CAGGCATCGTGATGGGTCC	IDT
SLC36A1	SLC36A1Rv	H. sapiens	TTGGCCGCTTCTATCACCTG	IDT
SLC38A1	SLC38A1Fw	H. sapiens	TTGGTGGTGAGGGGAGATTC	IDT
SLC38A1	SLC38A1Rv	H. sapiens	GAAGCTTGACACCCCTGTTAG	IDT
PHGDH	PHGDHFw	H. sapiens	GCAAATCTGCGGAAAGTGCT	IDT
PHGDH	PHGDHRv	H. sapiens	ATAAGGCCTTCACAGTCCTGC	IDT
SLC12A4	Hs_SLC12A4_1_SG	H. sapiens	QT00003787; LOT 462267267	Qiagen
SLC25A15	Hs_SLC25A15_1_SG	H. sapiens	QT00062867; LOT 457134948	Qiagen
SLC25A1	Hs_SLC25A1_1_SG	H. sapiens	QT00204862; LOT 353901508	Qiagen

Supplementary Table 5 - Antibodies							
Antibody/Dye	Species	Clone	Supplier	Ref. No.	LOT	Dilution	Application
PHGDH	Rabbit	Polyclonal	Cell Signaling Technology	13428	1	1;1000	WB
PSAT1	Rabbit	Polyclonal	Biotechne; Novus Biologicals	NBP1-32920	42242	1;1000	WB
PSPH	Rabbit	Polyclonal	Santa Cruz Biotechnology	sc-98683	B1909	1;1000	WB
SLC25A15	Rabbit	Polyclonal	Abcam	ab228604	GR3228856-8	1;1000	WB
UQCRFS1	Rabbit	Polyclonal	ProteinTech	18443-1-AP	00025413	1;1000	WB
GAPDH	Mouse	Monoclonal, clone 6C5	Merck, Sigma, Millipore	MAB374	2792998	1;2000	WB
ACTIN	Mouse	Monoclonal, clone C4	MERCK; Sigma; EMD	MAB1501	3166064	1;10000	WB
SLC12A4 (KCC1)	Rabbit	Polyclonal	Thermo Fisher Scientific	PA5-77471	XK3733383	1;1000	WB
SLC6A14	Rabbit	Polyclonal	Thermo Fisher Scientific	PA5104151	XJ3736482	1;1000	WB
Vinculin	Mouse	Monoclonal; 7F9	Santa Cruz	sc-73614	E1719	1;2000	WB
IRDye 680RD anti-Rabbit IgG	Donkey	Polyclonal	LI-COR	92568073	C70323-07	1;10000	WB
IRDye 800CW anti-Mouse IgG	Donkey	Polyclonal	LI-COR	92532212	C50422-05	1;10000	WB
Anti-mouse IgG, HRP-linked	n/a	n/a	Cell Signaling Technology	7076	38	1;2000	WB
Anti-rabbit IgG, HRP-linked	n/a	n/a	Cell Signaling Technology	7074	31	1;2000	WB
GFP	Chicken	Polyclonal	Abcam	ab13970	GR236651-22	1;2000	IF
DYKDDDDK (FLAG) Tag (9A3)	Mouse	Monoclonal	Cell Signaling Technology	8146	5	1;500	IF
SLC6A14	Rabbit	Polyclonal	St John's Laboratory	STJ112596	259600970101	1;500	IF
Alexa Fluor 488 anti-rabbit	Donkey	Polyclonal	Invitrogen	A21206	2289872	1;500	IF
Alexa Fluor 594 anti-mouse	Donkey	Polyclonal	Invitrogen	A21203	1918277	1;500	IF
Alexa Fluor 488 anti-chicken	Goat	Polyclonal	Invitrogen	A11039	2304258	1;500	IF
DAPI	n/a	n/a	Thermo Fisher Scientific	62248	VG3036772	1 µg mL^{-1}	IF
Alexa Fluor 647 Phalloidin	n/a	n/a	Invitrogen	A22287	1941485	1;100	IF
