# Supplementary material for: Phenotypic profiling of solute carriers characterizes serine transport in cancer
Source: Nat Metab. 2023 Dec 8;5(12):2148–68. doi: 10.1038/s42255-023-00936-2 (PMC10730406; doi:10.1038/s42255-023-00936-2)
Supplement: Supplementary file 2 — Reporting Summary [file 42255_2023_936_MOESM2_ESM.pdf]

nature portfolio

Corresponding author(s): Oliver D. K. Maddocks & Vasileios Papalazarou

Last updated by author(s): Sep 15, 2023

Reporting Summary

Nature Portfolio wishes to improve the reproducibility of the work that we publish. This form provides structure for consistency and transparency in reporting. For further information on Nature Portfolio policies, see our Editorial Policies and the Editorial Policy Checklist.

Statistics

For all statistical analyses, confirm that the following items are present in the figure legend, table legend, main text, or Methods section.

n/a	Confirmed	
☐	☒	The exact sample size (*n*) for each experimental group/condition, given as a discrete number and unit of measurement
☐	☒	A statement on whether measurements were taken from distinct samples or whether the same sample was measured repeatedly
☐	☒	The statistical test(s) used AND whether they are one- or two-sided<br>*Only common tests should be described solely by name; describe more complex techniques in the Methods section.*
☐	☒	A description of all covariates tested
☐	☒	A description of any assumptions or corrections, such as tests of normality and adjustment for multiple comparisons
☐	☒	A full description of the statistical parameters including central tendency (e.g. means) or other basic estimates (e.g. regression coefficient) AND variation (e.g. standard deviation) or associated estimates of uncertainty (e.g. confidence intervals)
☐	☒	For null hypothesis testing, the test statistic (e.g. *F*, *t*, *r*) with confidence intervals, effect sizes, degrees of freedom and *P* value noted<br>*Give P values as exact values whenever suitable.*
☒	☐	For Bayesian analysis, information on the choice of priors and Markov chain Monte Carlo settings
☒	☐	For hierarchical and complex designs, identification of the appropriate level for tests and full reporting of outcomes
☐	☒	Estimates of effect sizes (e.g. Cohen's *d*, Pearson's *r*), indicating how they were calculated

Our web collection on statistics for biologists contains articles on many of the points above.

Software and code

Policy information about availability of computer code

Data collection	Harmony High-Content Imaging and Analysis Software (Perkin-Elmer) version 4.1 and 4.9<br>Incucyte Software (Sartorius) v2021A and v2022A<br>Image Studio software (LI-COR) version 5.2<br>Xcalibur (ThermoFisher Scientific) version 2.2 and 4.2.47<br>QuantStudio Real-Time PCR Software (Applied Biosystems, ThermoFisher Scientific) version 1.7.2<br>MicroManager (version 2.0)
Data analysis	Columbus Image Data Storage and Analysis System (Perkin Elmer) version 2.8.0<br>ImageStudioLite (LI-COR) version 5.2.5<br>Fiji software (ImageJ) v2.0.0<br>Rstudio version 1.4.1717<br>Prism 9 (GraphPad Software) v.9.3.1<br>MSConvert (ProteoWizard) version 3.0.22133-5eed1a6<br>MZMine version 2.53<br>Incucyte S3 (Sartorius) v2021A and v2022A<br>Microsoft Excel (Microsoft) version 16.61<br>TraceFinder 4.1 EFS software (ThermoFisher)<br>MANIC software (version 3.0); in house-developed based on the software package GAVIN (doi: 10.1016/j.ab.2011.04.009)<br>Clustal Omega Multiple Sequence Alignment tool (EMBL-EBI)

For manuscripts utilizing custom algorithms or software that are central to the research but not yet described in published literature, software must be made available to editors and reviewers. We strongly encourage code deposition in a community repository (e.g. GitHub). See the Nature Portfolio guidelines for submitting code & software for further information.

Data

Policy information about availability of data

All manuscripts must include a data availability statement. This statement should provide the following information, where applicable:

- Accession codes, unique identifiers, or web links for publicly available datasets
- A description of any restrictions on data availability
- For clinical datasets or third party data, please ensure that the statement adheres to our policy

Associated raw data are provided as Source Data Files associated with each main or Extended Data figure. Original datasets, analyses and methodological details are available from the source data supplementary files and publicly available from https://researchdata.gla.ac.uk/. Information regarding experimental design and reagents can also be found in the Reporting Summary.

TCGA, TARGET and GTEx databases were accessed through the USCS Xena server and tools (http://xena.ucsc.edu/). Alphafold protein structure database was accessed from the following link https://alphafold.ebi.ac.uk/.

Human research participants

Policy information about studies involving human research participants and Sex and Gender in Research.

Reporting on sex and gender	N/A
Population characteristics	N/A
Recruitment	N/A
Ethics oversight	N/A

Note that full information on the approval of the study protocol must also be provided in the manuscript.

Field-specific reporting

Please select the one below that is the best fit for your research. If you are not sure, read the appropriate sections before making your selection.

☒ Life sciences ☐ Behavioural & social sciences ☐ Ecological, evolutionary & environmental sciences

For a reference copy of the document with all sections, see nature.com/documents/nr-reporting-summary-flat.pdf

Life sciences study design

All studies must disclose on these points even when the disclosure is negative.

Sample size	No statistical method was used to predetermine sample size. Sample sizes were estimated according to common practice for each experimental design and based on previous experience and pilot experiments to estimate variability. Our sample sizes are similar to those reported in previous publications (e.g. PMID 23242140, 28425994, 36115879, 32694686, 33831358)
Data exclusions	For in vivo experiments, a total of 67 mice were injected with cells – 21 NTC, 24 SLC6A14/12A4 and 22 SLC6A14/25A15. Prior to enrollment, four mice were excluded from the study as they either failed to develop tumours (one NTC and one SLC6A14/12A4) or had substantially delayed tumour growth / regressed (two SLC6A14/25A15).
Replication	Experimental findings were from minimum 3 biological replicates and / or independent experiments as stated in figure legends
Randomization	For in vitro experiments, unless otherwise stated, cells were randomly plated/treated/analysed during each experiment. For in vivo tumour experiments upon tumour formation (volume at ~70 mm3) mice were assigned into no-DOX (Control) and DOX (test condition) regimes in a manner ensuring a consistent starting volume across the groups.
Blinding	Investigators were blinded during metabolite data processing and analysis. We performed machine-learning based automated software analyses where applicable, e.g. cell number quantification for growth curves. For other molecular biology experiments (e.g. mRNA expression analysis), the same person was involved in experimental setup and analysis. For in vivo experiments, tumours were measured by staff blinded to the conditions and aims of the study.

Reporting for specific materials, systems and methods

We require information from authors about some types of materials, experimental systems and methods used in many studies. Here, indicate whether each material, system or method listed is relevant to your study. If you are not sure if a list item applies to your research, read the appropriate section before selecting a response.

Materials & experimental systems

n/a	Involved in the study
☐	☒ Antibodies
☐	☒ Eukaryotic cell lines
☒	☐ Palaeontology and archaeology
☐	☒ Animals and other organisms
☒	☐ Clinical data
☒	☐ Dual use research of concern

Methods

n/a	Involved in the study
☒	☐ ChIP-seq
☒	☐ Flow cytometry
☒	☐ MRI-based neuroimaging

Antibodies

Antibodies used

Antibody/Species/Clone/Supplier/REF/LOT/Dilution/Application
1. PHGDH/Rabbit/Polyclonal/Cell Signaling Technology/13428/1/1;1000/WB
2. PSAT1/Rabbit/Polyclonal/Biotechne; Novus Biologicals/NBP1-32920/42242/1;1000/WB
3. PSPH/Rabbit/Polyclonal/Santa Cruz Biotechnology/sc-98683/B1909/1;1000/WB
4. SLC25A15/Rabbit/Polyclonal/Abcam/ab228604/GR3228856-8/1;1000/WB
5. UQCRFS1/Rabbit/Polyclonal/ProteinTech/18443-1-AP/00025413/1;1000/WB
6. GAPDH/Mouse/Monoclonal; clone 6C5/Merck; Sigma; Millipore/MAB374/2792998/1;2000/WB
7. ACTIN/Mouse/Monoclonal, clone C4/Merck; Sigma; Millipore/MAB1501/3166064/1;10000/WB
8. SLC12A4 (KCC1)/Rabbit/Polyclonal/Thermo Fisher Scientific/PA5-77471/XK3733383/1:1000/WB
9. SLC6A14/Rabbit/Polyclonal/Thermo Fisher Scientific/PA5-104151/XJ3736482/1:1000/WB
10. Vinculin/Mouse/Monoclonal; clone 7F9/Santa Cruz/sc-73614/E1719/1:2000/WB
11. IRDye 680RD anti-Rabbit IgG/Donkey/Polyclonal/LI-COR/92568073/C70323-07/1;10000/WB
12. IRDye 800CW anti-Mouse IgG/Donkey/Polyclonal/LI-COR/92532212/C50422-05/1;10000/WB
13. GFP/Chicken/Polyclonal/Abcam/ab13970/GR236651-22/1;2000/IF
14. DYKDDDDK (FLAG) Tag (9A3)/Mouse/Monoclonal clone 9A3/Cell Signaling Technology/8146.5/1;500/IF
15. SLC6A14/Rabbit/Polyclonal/St John's Laboratory/STJ112596/259600970101/1;500/IF
16. Alexa Fluor 488 anti-rabbit/Donkey/Polyclonal/Invitrogen/A21206/2289872/1;500/IF
17. Alexa Fluor 594 anti-mouse/Donkey/Polyclonal/Invitrogen/A21203/1918277/1;500/IF
18. Alexa Fluor 488 anti-chicken/Goat/Polyclonal/Invitrogen/A11039/2304258/1;500/IF
19. DAPI///Thermo Fisher Scientific/62248/VG3036772/1µg/mL/IF
20. Alexa Fluor 647 Phalloidin///Invitrogen/A22287/1941485/1;100/IF
21. anti-rabbit HRP linked secondary antibody/Goat/Polyclonal/Cell Signaling Technology/7074/31/1:1000/WB
22. anti-mouse HRP linked secondary antibody/Horse/Polyclonal/Cell Signaling Technology/7076/38/1:1000/WB

Validation

1. PHGDH (CST;13428) - Validated by manufacturer for Western blot with analysis of extracts from 293, HeLa, and NIH/3T3 cells using and in-house with PHGDH knock-out cell lines
2. PSAT1 (Novus Biologicals; NBP1-32920) - Validated by manufacturer (knockdown) and used routinely in host lab
3. PSPH (Santa Cruz Biotechnology; sc-98683) - Validated by manufacturer (overexpression) and used routinely in host lab
4. SLC25A15 (Abcam; ab228604) - Validated by manufacturer for Western blot using Raji whole cell lysates and in-house by protein analysis of lysates from SLC25A15 knockdown, knockout and SLC25A15-overexpressing cells
5. UQCRFS1 (ProteinTech;18443-1-AP) - Validated by manufacturer for Western blot using MCF-7 cells, mouse brain tissue, mouse heart tissue, HEK-293 cells, HeLa cells, A2780 cells, rat heart tissue extracts.
6. GAPDH (Merck; MAB374) - Validated by manufacturer and used routinely in host lab
7. ACTIN (Merck; MAB1501) - Validated by manufacturer and used routinely in host lab
8. GFP (Abcam; ab13970) - Validated by manufacturer for Western blot by lysates of GFP-positive cells and in-house by expression of GFP positive and GFP negative cell lines
9. DYKDDDDK (FLAG) Tag (CST; 8146) - Validated by manufacturer and in-house by expression of FLAG positive and FLAG negative cell lines
10. SLC6A14 (StJohn'sLaboratory; STJ112596) - Validated in-house with overexpression and knockout cell lines as well as serial dilution experiments to detect optimal signal-to-noise ratio for immunofluorescence.
11. SLC12A4 (Thermo Fisher Scientific; PA5-77471) - Validated in-house by protein analysis of lysates from SLC12A4 knockdown, knockout and SLC12A4-overexpression cells.
12. SLC6A14 (Thermo Fisher Scientific; PA5-104151) - Validated in-house by protein analysis of lysates from SLC6A14 knockdown, knockout and SLC6A14-overexpression cells.
13. Vinculin (Santa Cruz;sc-73614) - Validated by manufacturer and used routinely in host lab

Eukaryotic cell lines

Policy information about cell lines and Sex and Gender in Research

Cell line source(s)

HCT116, DLD-1, MCF-7, MDA-MB-231, HEK293 H. sapiens cells and COS-7 C. aethiops cells were obtained from ATCC. HCT116 p21-/- were a gift of B. Vogelstein.

Authentication

Human cell lines were authenticated by STR profiling using Promega GenePrint 10

Mycoplasma contamination	Cell lines were routinely assessed for mycoplasma using Mycoalert (Lonza) and tested negative prior to any experimental applications.
Commonly misidentified lines (See ICLAC register)	No cell lines used in this study were found in the database of commonly misidentified cell lines maintained by ICLAC

Animals and other research organisms

Policy information about studies involving animals; ARRIVE guidelines recommended for reporting animal research, and Sex and Gender in Research

Laboratory animals	Animals used in this study were CD-1-nude female mice, 7-8-week old (Charles River Laboratories, UK).
Wild animals	No wild animals were used
Reporting on sex	All animals used in this study were female mice
Field-collected samples	No field-collected samples were used
Ethics oversight	All in vivo work was carried out in compliance with the Animals (Scientific Procedures) Act 1986 and the EU Directive 2010 (PPLs 70/8645 and PP6345023) and was sanctioned by the local animal welfare ethical review board (University of Glasgow).

Note that full information on the approval of the study protocol must also be provided in the manuscript.
