# Supplementary figures and images for: Phenotypic profiling of solute carriers characterizes serine transport in cancer
Source: Nat Metab. 2023 Dec 8;5(12):2148–68. doi: 10.1038/s42255-023-00936-2 (PMC10730406; doi:10.1038/s42255-023-00936-2)

Extended Data Figure 2a

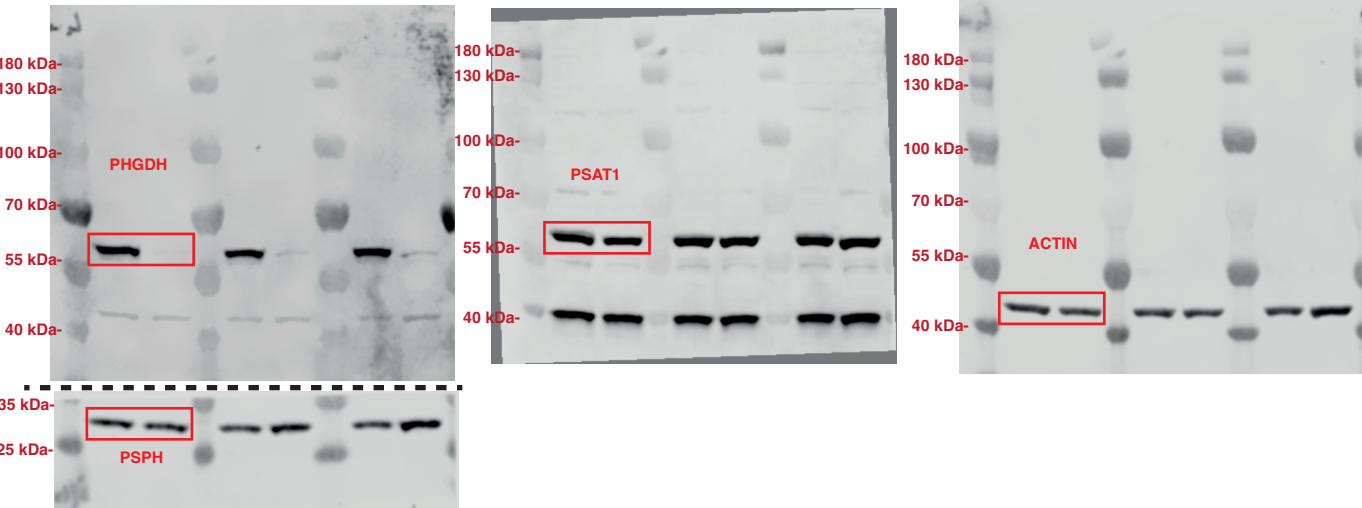

Extended Data Figure 3i

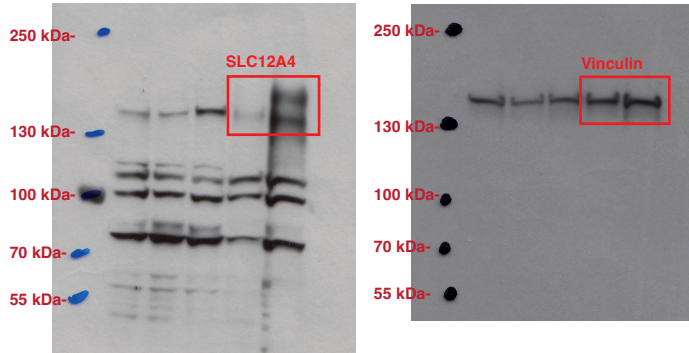

Extended Data Figure 4c

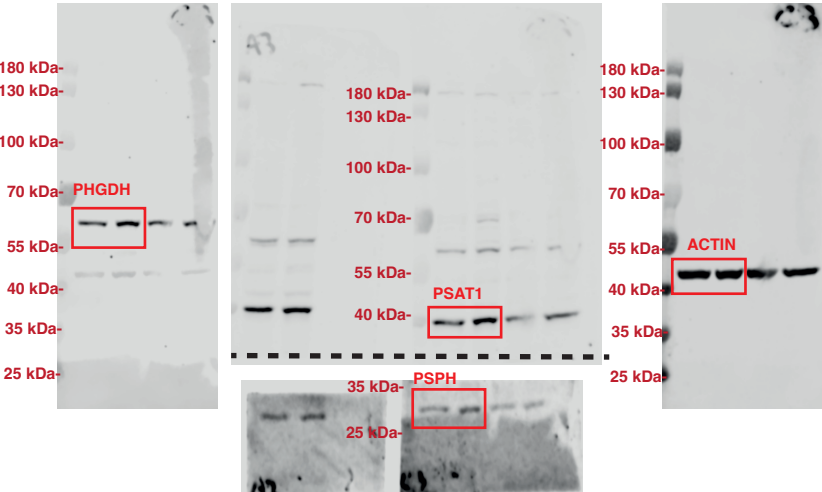

Supplement: Supplementary file 18 — Unprocessed blots. [file 42255_2023_936_MOESM18_ESM.pdf]
