# Supplementary material for: Phenotypic profiling of solute carriers characterizes serine transport in cancer
Source: Nat Metab. 2023 Dec 8;5(12):2148–68. doi: 10.1038/s42255-023-00936-2 (PMC10730406; doi:10.1038/s42255-023-00936-2)
Supplement: Supplementary file 17 — Unprocessed blots. [file 42255_2023_936_MOESM17_ESM.pdf]

Figure 2b

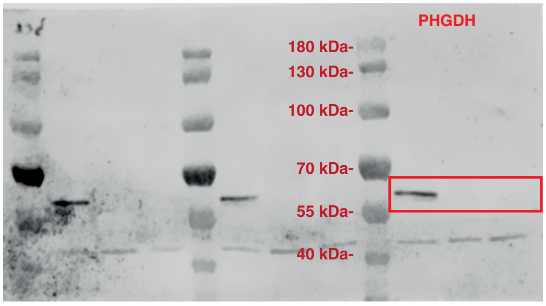

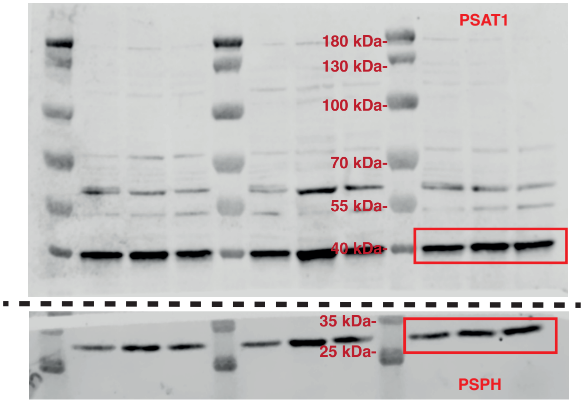

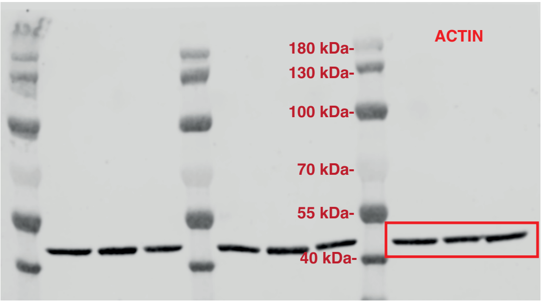

Figure 4a

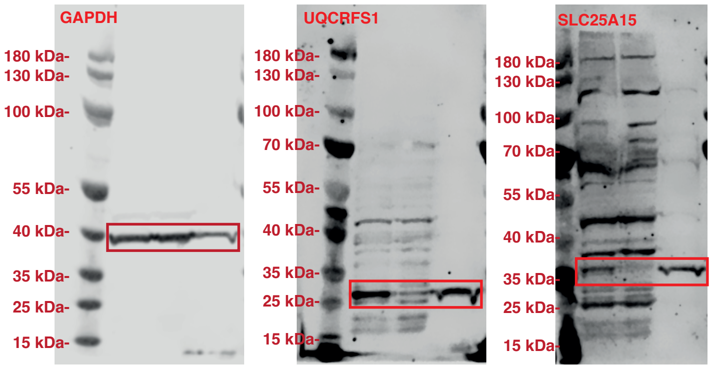

Figure 4e

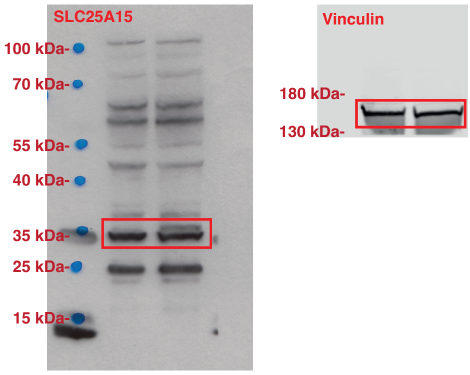

Extended Data Fig. 1d

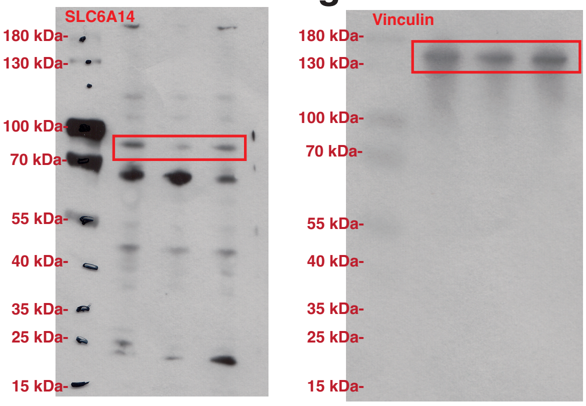

Extended Data Fig. 1e

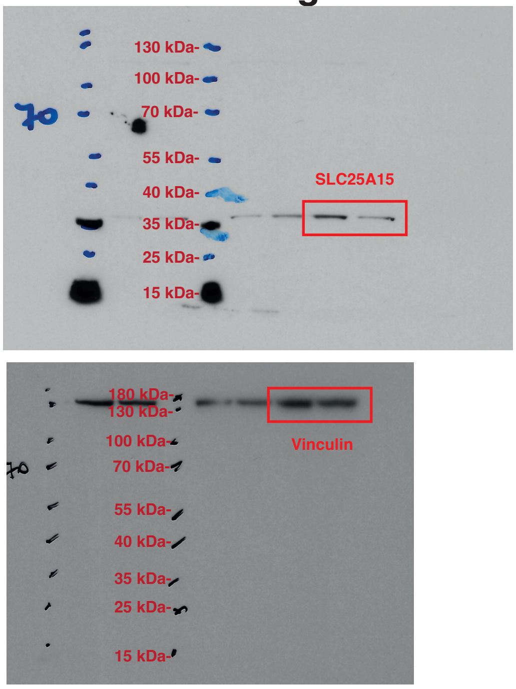

Extended Data Fig. 1f

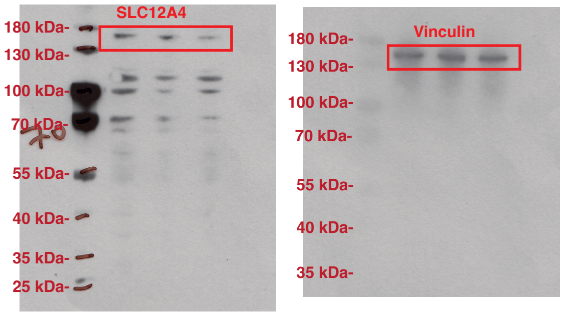

Source Data File 1
